# Supplementary material for: Research on coupling coordination between construction industry innovation and region economic development in China
Source: PLoS One. 2024 Aug 5;19(8):e0308127. doi: 10.1371/journal.pone.0308127 (PMC11299830; doi:10.1371/journal.pone.0308127)
Supplement: S1 Data — (DOCX) [file pone.0308127.s001.docx]

## S1 CII Standardization Data

S1-1 CII Standardization Data in 2012

| **Scope of research** | **C1** | **C2** | **C3** | **C4** | **C5** | **C6** | **C7** | **C8** | **C9** | **C10** | **C11** | **C12** | **C13** | **C14** | **C15** | **C16** | **C17** | **C18** | **C19** |
| --- | --- | --- | --- | --- | --- | --- | --- | --- | --- | --- | --- | --- | --- | --- | --- | --- | --- | --- | --- |
| Beijing | 0.720 | 0.061 | 0.190 | 0.330 | 0.467 | 0.400 | 0.700 | 0.180 | 0.820 | 0.930 | 0.180 | 1.000 | 0.426 | 0.670 | 1.000 | 0.000 | 1.000 | 0.187 | 1.000 |
| Tianjin | 0.340 | 0.039 | 0.100 | 0.130 | 0.068 | 0.176 | 0.300 | 1.000 | 0.060 | 0.020 | 0.270 | 0.700 | 0.196 | 0.500 | 0.690 | 0.200 | 0.197 | 0.073 | 0.640 |
| Hebei | 0.280 | 0.178 | 0.170 | 0.200 | 0.501 | 0.275 | 0.100 | 0.090 | 0.320 | 0.240 | 0.170 | 0.152 | 0.167 | 0.000 | 0.230 | 0.200 | 0.281 | 0.056 | 0.250 |
| Shanxi | 0.030 | 0.085 | 0.090 | 0.090 | 0.361 | 0.219 | 0.000 | 0.150 | 0.090 | 0.110 | 0.060 | 0.165 | 0.310 | 0.330 | 0.080 | 0.000 | 0.115 | 0.026 | 0.120 |
| Inner Mongolia | 0.100 | 0.045 | 0.080 | 0.110 | 0.240 | 0.091 | 0.100 | 0.130 | 0.040 | 0.030 | 0.040 | 0.135 | 0.314 | 0.000 | 0.080 | 0.000 | 0.105 | 0.011 | 0.080 |
| Liaoning | 0.380 | 0.266 | 0.320 | 0.350 | 0.472 | 0.629 | 0.100 | 0.070 | 0.170 | 0.170 | 0.170 | 0.363 | 0.367 | 0.170 | 0.150 | 0.400 | 0.443 | 0.078 | 0.150 |
| Jilin | 0.170 | 0.067 | 0.080 | 0.100 | 0.303 | 0.177 | 0.200 | 0.090 | 0.090 | 0.020 | 0.040 | 0.191 | 0.441 | 0.000 | 0.150 | 0.000 | 0.218 | 0.021 | 0.170 |
| Heilongjiang | 0.210 | 0.062 | 0.080 | 0.080 | 0.369 | 0.222 | 0.100 | 0.120 | 0.020 | 0.010 | 0.090 | 0.200 | 0.672 | 0.000 | 0.000 | 0.200 | 0.325 | 0.075 | 0.030 |
| Shanghai | 0.340 | 0.114 | 0.160 | 0.220 | 0.301 | 0.329 | 1.000 | 0.140 | 0.370 | 0.520 | 0.290 | 0.551 | 0.398 | 0.330 | 0.770 | 0.400 | 0.628 | 0.190 | 0.390 |
| Jiangsu | 1.000 | 1.000 | 1.000 | 1.000 | 1.000 | 1.000 | 0.100 | 0.180 | 0.320 | 0.200 | 1.000 | 0.403 | 0.577 | 0.000 | 0.230 | 1.000 | 0.906 | 1.000 | 0.080 |
| Zhejiang | 0.280 | 0.866 | 0.840 | 0.630 | 0.554 | 0.715 | 0.300 | 0.010 | 0.310 | 0.180 | 0.800 | 0.346 | 0.353 | 0.330 | 0.150 | 0.800 | 0.396 | 0.698 | 0.100 |
| Anhui | 0.280 | 0.226 | 0.200 | 0.200 | 0.369 | 0.294 | 0.000 | 0.050 | 0.580 | 0.220 | 0.270 | 0.242 | 0.235 | 0.170 | 0.000 | 0.400 | 0.311 | 0.160 | 0.080 |
| Fujian | 0.240 | 0.247 | 0.320 | 0.200 | 0.259 | 0.328 | 0.100 | 0.020 | 0.060 | 0.030 | 0.200 | 0.204 | 0.280 | 0.000 | 0.080 | 0.200 | 0.152 | 0.113 | 0.000 |
| Jiangxi | 0.100 | 0.141 | 0.110 | 0.130 | 0.235 | 0.160 | 0.000 | 0.020 | 0.010 | 0.000 | 0.070 | 0.120 | 0.340 | 0.170 | 0.080 | 0.200 | 0.202 | 0.029 | 0.120 |
| Shandong | 0.410 | 0.372 | 0.350 | 0.440 | 0.802 | 0.642 | 0.200 | 0.060 | 0.440 | 0.360 | 0.680 | 0.399 | 1.000 | 0.170 | 0.380 | 0.400 | 0.422 | 0.279 | 0.030 |
| Henan | 0.210 | 0.303 | 0.250 | 0.320 | 0.493 | 0.535 | 0.200 | 0.070 | 0.340 | 0.350 | 0.210 | 0.154 | 0.226 | 0.000 | 0.000 | 0.000 | 0.415 | 0.099 | 0.130 |
| Hubei | 0.590 | 0.227 | 0.280 | 0.380 | 0.388 | 0.307 | 0.100 | 0.160 | 1.000 | 1.000 | 0.180 | 0.272 | 0.313 | 0.500 | 0.080 | 0.800 | 0.625 | 0.090 | 0.360 |
| Hunan | 0.410 | 0.156 | 0.170 | 0.200 | 0.327 | 0.220 | 0.100 | 0.070 | 0.530 | 0.360 | 0.170 | 0.208 | 0.573 | 0.000 | 0.080 | 0.000 | 0.394 | 0.086 | 0.110 |
| Guangdong | 0.450 | 0.254 | 0.330 | 0.390 | 0.750 | 0.523 | 0.300 | 0.100 | 0.370 | 0.240 | 0.840 | 0.360 | 0.768 | 1.000 | 0.540 | 1.000 | 0.567 | 0.569 | 0.260 |
| Guangxi | 0.100 | 0.086 | 0.070 | 0.040 | 0.245 | 0.107 | 0.000 | 0.010 | 0.030 | 0.210 | 0.080 | 0.114 | 0.537 | 0.000 | 0.230 | 0.000 | 0.211 | 0.021 | 0.120 |
| Hainan | 0.000 | 0.003 | 0.010 | 0.010 | 0.064 | 0.000 | 0.100 | 0.000 | 0.000 | 0.000 | 0.010 | 0.045 | 0.000 | 0.000 | 0.000 | 0.000 | 0.029 | 0.004 | 0.430 |
| Chongqing | 0.240 | 0.187 | 0.170 | 0.220 | 0.148 | 0.284 | 0.100 | 0.010 | 0.010 | 0.010 | 0.110 | 0.212 | 0.365 | 0.170 | 0.080 | 0.000 | 0.262 | 0.075 | 0.080 |
| Sichuan | 0.240 | 0.293 | 0.250 | 0.280 | 0.480 | 0.482 | 0.200 | 0.030 | 0.130 | 0.100 | 0.220 | 0.228 | 0.352 | 0.170 | 0.150 | 0.600 | 0.486 | 0.156 | 0.070 |
| Guizhou | 0.000 | 0.043 | 0.040 | 0.020 | 0.108 | 0.058 | 0.000 | 0.010 | 0.010 | 0.000 | 0.040 | 0.069 | 0.172 | 0.170 | 0.080 | 0.000 | 0.113 | 0.022 | 0.190 |
| Yunnan | 0.140 | 0.097 | 0.080 | 0.110 | 0.225 | 0.226 | 0.100 | 0.100 | 0.010 | 0.000 | 0.040 | 0.069 | 0.524 | 0.170 | 0.080 | 0.000 | 0.155 | 0.021 | 0.080 |
| Tibet | 0.000 | 0.000 | 0.000 | 0.000 | 0.000 | 0.005 | 0.000 | 0.070 | 0.000 | 0.000 | 0.000 | 0.000 | 0.038 | 0.000 | 0.000 | 0.000 | 0.000 | 0.000 | 0.080 |
| Shaanxi | 0.520 | 0.103 | 0.120 | 0.130 | 0.509 | 0.141 | 0.000 | 0.090 | 0.230 | 0.180 | 0.120 | 0.333 | 0.154 | 0.170 | 0.080 | 0.400 | 0.444 | 0.055 | 0.260 |
| Gansu | 0.170 | 0.071 | 0.060 | 0.060 | 0.219 | 0.126 | 0.100 | 0.050 | 0.010 | 0.010 | 0.040 | 0.163 | 0.354 | 0.170 | 0.000 | 0.000 | 0.128 | 0.013 | 0.070 |
| Qinghai | 0.000 | 0.011 | 0.010 | 0.010 | 0.089 | 0.038 | 0.000 | 0.120 | 0.010 | 0.000 | 0.000 | 0.114 | 0.011 | 0.000 | 0.080 | 0.000 | 0.014 | 0.001 | 0.060 |
| Ningxia | 0.000 | 0.007 | 0.020 | 0.010 | 0.084 | 0.051 | 0.000 | 0.110 | 0.090 | 0.030 | 0.030 | 0.114 | 0.051 | 0.000 | 0.000 | 0.000 | 0.044 | 0.003 | 0.060 |
| Xinjiang | 0.000 | 0.036 | 0.070 | 0.040 | 0.158 | 0.106 | 0.100 | 0.100 | 0.020 | 0.010 | 0.020 | 0.054 | 0.310 | 0.170 | 0.230 | 0.000 | 0.099 | 0.012 | 0.060 |

S1-2 CII Standardization Data in 2013

| **Scope of research** | **C1** | **C2** | **C3** | **C4** | **C5** | **C6** | **C7** | **C8** | **C9** | **C10** | **C11** | **C12** | **C13** | **C14** | **C15** | **C16** | **C17** | **C18** | **C19** |
| --- | --- | --- | --- | --- | --- | --- | --- | --- | --- | --- | --- | --- | --- | --- | --- | --- | --- | --- | --- |
| Beijing | 0.700 | 0.060 | 0.160 | 0.390 | 0.450 | 0.377 | 0.750 | 0.260 | 0.800 | 0.850 | 0.210 | 1.000 | 0.459 | 0.670 | 1.000 | 0.059 | 1.000 | 0.261 | 1.000 |
| Tianjin | 0.330 | 0.039 | 0.110 | 0.150 | 0.099 | 0.176 | 0.130 | 1.000 | 0.060 | 0.020 | 0.270 | 0.754 | 0.179 | 0.170 | 0.360 | 0.529 | 0.230 | 0.103 | 0.490 |
| Hebei | 0.270 | 0.152 | 0.140 | 0.180 | 0.469 | 0.262 | 0.130 | 0.160 | 0.330 | 0.240 | 0.160 | 0.165 | 0.149 | 0.000 | 0.140 | 0.235 | 0.267 | 0.075 | 0.320 |
| Shanxi | 0.070 | 0.084 | 0.080 | 0.090 | 0.338 | 0.227 | 0.000 | 0.250 | 0.090 | 0.110 | 0.060 | 0.189 | 0.289 | 0.000 | 0.210 | 0.000 | 0.110 | 0.035 | 0.100 |
| Inner Mongolia | 0.100 | 0.047 | 0.080 | 0.120 | 0.231 | 0.089 | 0.130 | 0.180 | 0.040 | 0.030 | 0.040 | 0.141 | 0.293 | 0.170 | 0.000 | 0.059 | 0.109 | 0.016 | 0.060 |
| Liaoning | 0.370 | 0.254 | 0.300 | 0.350 | 0.447 | 0.642 | 0.130 | 0.140 | 0.170 | 0.170 | 0.160 | 0.383 | 0.338 | 0.000 | 0.430 | 0.294 | 0.450 | 0.090 | 0.220 |
| Jilin | 0.200 | 0.055 | 0.070 | 0.100 | 0.283 | 0.190 | 0.000 | 0.140 | 0.080 | 0.020 | 0.130 | 0.186 | 0.440 | 0.000 | 0.000 | 0.000 | 0.246 | 0.025 | 0.190 |
| Heilongjiang | 0.200 | 0.065 | 0.060 | 0.070 | 0.349 | 0.207 | 0.250 | 0.230 | 0.020 | 0.010 | 0.090 | 0.208 | 0.698 | 0.000 | 0.070 | 0.118 | 0.305 | 0.082 | 0.010 |
| Shanghai | 0.330 | 0.103 | 0.130 | 0.210 | 0.269 | 0.297 | 1.000 | 0.200 | 0.380 | 0.480 | 0.280 | 0.576 | 0.367 | 0.500 | 0.710 | 1.000 | 0.635 | 0.203 | 0.300 |
| Jiangsu | 1.000 | 1.000 | 1.000 | 1.000 | 1.000 | 1.000 | 0.250 | 0.170 | 0.330 | 0.200 | 1.000 | 0.418 | 0.576 | 0.170 | 0.430 | 0.941 | 0.924 | 1.000 | 0.070 |
| Zhejiang | 0.300 | 0.878 | 0.780 | 0.590 | 0.557 | 0.725 | 0.500 | 0.040 | 0.320 | 0.170 | 0.870 | 0.358 | 0.311 | 0.000 | 0.210 | 0.353 | 0.377 | 0.844 | 0.120 |
| Anhui | 0.300 | 0.221 | 0.220 | 0.200 | 0.355 | 0.291 | 0.000 | 0.110 | 0.610 | 0.230 | 0.300 | 0.268 | 0.318 | 0.330 | 0.140 | 0.118 | 0.324 | 0.203 | 0.100 |
| Fujian | 0.270 | 0.284 | 0.320 | 0.200 | 0.291 | 0.344 | 0.000 | 0.050 | 0.050 | 0.030 | 0.210 | 0.210 | 0.296 | 0.000 | 0.140 | 0.235 | 0.158 | 0.156 | 0.010 |
| Jiangxi | 0.130 | 0.158 | 0.120 | 0.140 | 0.219 | 0.164 | 0.250 | 0.070 | 0.010 | 0.000 | 0.090 | 0.126 | 0.349 | 0.000 | 0.000 | 0.059 | 0.205 | 0.041 | 0.130 |
| Shandong | 0.400 | 0.373 | 0.380 | 0.460 | 0.763 | 0.621 | 0.130 | 0.130 | 0.440 | 0.350 | 0.660 | 0.413 | 1.000 | 0.170 | 0.290 | 0.353 | 0.439 | 0.321 | 0.070 |
| Henan | 0.200 | 0.318 | 0.270 | 0.340 | 0.468 | 0.559 | 0.130 | 0.130 | 0.380 | 0.350 | 0.230 | 0.158 | 0.246 | 0.000 | 0.290 | 0.118 | 0.391 | 0.123 | 0.110 |
| Hubei | 0.600 | 0.230 | 0.310 | 0.420 | 0.365 | 0.342 | 0.380 | 0.240 | 1.000 | 1.000 | 0.200 | 0.278 | 0.315 | 0.330 | 0.290 | 0.353 | 0.619 | 0.120 | 0.410 |
| Hunan | 0.430 | 0.175 | 0.230 | 0.210 | 0.315 | 0.217 | 0.000 | 0.140 | 0.500 | 0.360 | 0.170 | 0.208 | 0.541 | 0.000 | 0.000 | 0.000 | 0.402 | 0.101 | 0.050 |
| Guangdong | 0.430 | 0.260 | 0.340 | 0.400 | 0.715 | 0.539 | 0.250 | 0.190 | 0.330 | 0.230 | 0.840 | 0.381 | 0.726 | 1.000 | 0.290 | 0.588 | 0.553 | 0.711 | 0.230 |
| Guangxi | 0.100 | 0.094 | 0.070 | 0.040 | 0.233 | 0.102 | 0.130 | 0.000 | 0.030 | 0.200 | 0.060 | 0.111 | 0.521 | 0.170 | 0.000 | 0.000 | 0.211 | 0.032 | 0.130 |
| Hainan | 0.000 | 0.003 | 0.010 | 0.010 | 0.059 | 0.000 | 0.250 | 0.030 | 0.000 | 0.000 | 0.020 | 0.038 | 0.001 | 0.170 | 0.070 | 0.059 | 0.033 | 0.005 | 0.360 |
| Chongqing | 0.230 | 0.206 | 0.200 | 0.250 | 0.135 | 0.271 | 0.380 | 0.040 | 0.010 | 0.010 | 0.120 | 0.201 | 0.369 | 0.330 | 0.000 | 0.059 | 0.255 | 0.103 | 0.100 |
| Sichuan | 0.300 | 0.315 | 0.240 | 0.270 | 0.474 | 0.460 | 0.630 | 0.140 | 0.140 | 0.100 | 0.210 | 0.231 | 0.362 | 0.170 | 0.140 | 0.235 | 0.488 | 0.192 | 0.050 |
| Guizhou | 0.000 | 0.050 | 0.040 | 0.030 | 0.119 | 0.058 | 0.130 | 0.050 | 0.020 | 0.000 | 0.030 | 0.058 | 0.178 | 0.000 | 0.000 | 0.118 | 0.123 | 0.033 | 0.220 |
| Yunnan | 0.130 | 0.100 | 0.100 | 0.130 | 0.209 | 0.231 | 0.000 | 0.210 | 0.010 | 0.000 | 0.040 | 0.064 | 0.483 | 0.170 | 0.070 | 0.000 | 0.176 | 0.028 | 0.070 |
| Tibet | 0.000 | 0.000 | 0.000 | 0.000 | 0.000 | 0.001 | 0.000 | 0.140 | 0.000 | 0.000 | 0.000 | 0.000 | 0.020 | 0.000 | 0.000 | 0.000 | 0.000 | 0.000 | 0.090 |
| Shaanxi | 0.500 | 0.121 | 0.150 | 0.150 | 0.547 | 0.147 | 0.250 | 0.200 | 0.250 | 0.170 | 0.130 | 0.351 | 0.181 | 0.170 | 0.140 | 0.176 | 0.435 | 0.086 | 0.220 |
| Gansu | 0.170 | 0.070 | 0.060 | 0.060 | 0.214 | 0.127 | 0.000 | 0.100 | 0.010 | 0.010 | 0.040 | 0.156 | 0.324 | 0.000 | 0.000 | 0.000 | 0.142 | 0.019 | 0.100 |
| Qinghai | 0.000 | 0.011 | 0.010 | 0.010 | 0.083 | 0.034 | 0.130 | 0.250 | 0.010 | 0.000 | 0.000 | 0.098 | 0.000 | 0.170 | 0.000 | 0.000 | 0.011 | 0.002 | 0.090 |
| Ningxia | 0.000 | 0.012 | 0.010 | 0.010 | 0.078 | 0.047 | 0.000 | 0.150 | 0.090 | 0.020 | 0.020 | 0.116 | 0.038 | 0.000 | 0.000 | 0.118 | 0.040 | 0.005 | 0.000 |
| Xinjiang | 0.000 | 0.043 | 0.080 | 0.040 | 0.143 | 0.108 | 0.130 | 0.200 | 0.020 | 0.010 | 0.020 | 0.049 | 0.293 | 0.000 | 0.000 | 0.059 | 0.101 | 0.020 | 0.060 |

S1-3 CII Standardization Data in 2014

| **Scope of research** | **C1** | **C2** | **C3** | **C4** | **C5** | **C6** | **C7** | **C8** | **C9** | **C10** | **C11** | **C12** | **C13** | **C14** | **C15** | **C16** | **C17** | **C18** | **C19** |
| --- | --- | --- | --- | --- | --- | --- | --- | --- | --- | --- | --- | --- | --- | --- | --- | --- | --- | --- | --- |
| Beijing | 0.710 | 0.061 | 0.190 | 0.440 | 0.435 | 0.326 | 0.800 | 0.410 | 0.740 | 0.800 | 0.170 | 1.000 | 0.567 | 0.670 | 1.000 | 0.250 | 1.000 | 0.373 | 1.000 |
| Tianjin | 0.320 | 0.039 | 0.110 | 0.160 | 0.126 | 0.183 | 0.100 | 1.000 | 0.060 | 0.020 | 0.280 | 0.780 | 0.183 | 0.170 | 0.360 | 0.240 | 0.242 | 0.131 | 0.620 |
| Hebei | 0.290 | 0.142 | 0.130 | 0.160 | 0.442 | 0.264 | 0.000 | 0.290 | 0.360 | 0.230 | 0.160 | 0.188 | 0.172 | 0.170 | 0.140 | 0.310 | 0.246 | 0.100 | 0.540 |
| Shanxi | 0.060 | 0.076 | 0.080 | 0.090 | 0.319 | 0.249 | 0.000 | 0.440 | 0.090 | 0.100 | 0.050 | 0.191 | 0.238 | 0.000 | 0.210 | 0.130 | 0.118 | 0.041 | 0.190 |
| Inner Mongolia | 0.130 | 0.039 | 0.050 | 0.070 | 0.222 | 0.091 | 0.000 | 0.290 | 0.040 | 0.030 | 0.040 | 0.142 | 0.275 | 0.000 | 0.000 | 0.050 | 0.105 | 0.019 | 0.130 |
| Liaoning | 0.390 | 0.214 | 0.220 | 0.260 | 0.425 | 0.662 | 0.200 | 0.280 | 0.170 | 0.140 | 0.160 | 0.364 | 0.369 | 0.000 | 0.430 | 0.070 | 0.427 | 0.097 | 0.310 |
| Jilin | 0.230 | 0.056 | 0.060 | 0.110 | 0.266 | 0.231 | 0.000 | 0.260 | 0.070 | 0.020 | 0.040 | 0.201 | 0.441 | 0.170 | 0.000 | 0.070 | 0.231 | 0.033 | 0.270 |
| Heilongjiang | 0.190 | 0.043 | 0.050 | 0.050 | 0.331 | 0.189 | 0.200 | 0.410 | 0.020 | 0.010 | 0.080 | 0.205 | 0.684 | 0.000 | 0.070 | 0.040 | 0.324 | 0.076 | 0.040 |
| Shanghai | 0.320 | 0.094 | 0.130 | 0.200 | 0.241 | 0.309 | 1.000 | 0.320 | 0.360 | 0.450 | 0.260 | 0.598 | 0.368 | 0.500 | 0.710 | 0.390 | 0.640 | 0.252 | 0.650 |
| Jiangsu | 1.000 | 1.000 | 1.000 | 1.000 | 1.000 | 1.000 | 0.400 | 0.170 | 0.340 | 0.190 | 1.000 | 0.436 | 0.690 | 0.170 | 0.430 | 1.000 | 0.950 | 1.000 | 0.210 |
| Zhejiang | 0.290 | 0.919 | 0.790 | 0.590 | 0.559 | 0.666 | 0.300 | 0.110 | 0.330 | 0.170 | 0.860 | 0.383 | 0.367 | 0.000 | 0.210 | 0.270 | 0.337 | 0.943 | 0.240 |
| Anhui | 0.290 | 0.215 | 0.210 | 0.190 | 0.343 | 0.299 | 0.100 | 0.220 | 0.630 | 0.220 | 0.280 | 0.284 | 0.351 | 0.330 | 0.140 | 0.110 | 0.327 | 0.241 | 0.270 |
| Fujian | 0.350 | 0.316 | 0.340 | 0.240 | 0.319 | 0.404 | 0.000 | 0.130 | 0.060 | 0.030 | 0.210 | 0.222 | 0.349 | 0.170 | 0.140 | 0.100 | 0.161 | 0.189 | 0.000 |
| Jiangxi | 0.130 | 0.166 | 0.130 | 0.160 | 0.205 | 0.176 | 0.100 | 0.180 | 0.010 | 0.000 | 0.080 | 0.138 | 0.332 | 0.000 | 0.000 | 0.100 | 0.209 | 0.068 | 0.240 |
| Shandong | 0.420 | 0.339 | 0.360 | 0.430 | 0.728 | 0.632 | 0.200 | 0.260 | 0.420 | 0.340 | 0.650 | 0.439 | 1.000 | 0.170 | 0.290 | 0.550 | 0.471 | 0.364 | 0.150 |
| Henan | 0.190 | 0.303 | 0.260 | 0.320 | 0.446 | 0.561 | 0.200 | 0.240 | 0.380 | 0.340 | 0.240 | 0.172 | 0.258 | 0.000 | 0.290 | 0.190 | 0.399 | 0.166 | 0.250 |
| Hubei | 0.610 | 0.243 | 0.280 | 0.400 | 0.344 | 0.346 | 0.600 | 0.380 | 1.000 | 1.000 | 0.190 | 0.295 | 0.317 | 0.330 | 0.290 | 0.280 | 0.628 | 0.141 | 0.920 |
| Hunan | 0.480 | 0.173 | 0.210 | 0.210 | 0.305 | 0.221 | 0.100 | 0.270 | 0.490 | 0.360 | 0.180 | 0.222 | 0.570 | 0.170 | 0.000 | 0.170 | 0.398 | 0.133 | 0.170 |
| Guangdong | 0.480 | 0.251 | 0.320 | 0.380 | 0.684 | 0.545 | 0.500 | 0.340 | 0.310 | 0.220 | 0.810 | 0.398 | 0.827 | 1.000 | 0.290 | 0.700 | 0.510 | 0.900 | 0.500 |
| Guangxi | 0.100 | 0.096 | 0.080 | 0.050 | 0.222 | 0.105 | 0.000 | 0.000 | 0.030 | 0.180 | 0.060 | 0.108 | 0.538 | 0.170 | 0.000 | 0.120 | 0.207 | 0.048 | 0.200 |
| Hainan | 0.000 | 0.006 | 0.010 | 0.010 | 0.054 | 0.000 | 0.000 | 0.090 | 0.000 | 0.000 | 0.020 | 0.045 | 0.000 | 0.170 | 0.070 | 0.020 | 0.033 | 0.007 | 0.520 |
| Chongqing | 0.230 | 0.209 | 0.210 | 0.280 | 0.123 | 0.275 | 0.100 | 0.110 | 0.010 | 0.010 | 0.150 | 0.214 | 0.323 | 0.330 | 0.000 | 0.070 | 0.267 | 0.121 | 0.240 |
| Sichuan | 0.320 | 0.286 | 0.220 | 0.230 | 0.469 | 0.430 | 0.200 | 0.330 | 0.140 | 0.100 | 0.210 | 0.248 | 0.482 | 0.170 | 0.140 | 0.100 | 0.507 | 0.235 | 0.100 |
| Guizhou | 0.000 | 0.054 | 0.040 | 0.030 | 0.129 | 0.063 | 0.000 | 0.130 | 0.010 | 0.000 | 0.030 | 0.066 | 0.226 | 0.000 | 0.000 | 0.070 | 0.122 | 0.050 | 0.330 |
| Yunnan | 0.160 | 0.098 | 0.090 | 0.120 | 0.194 | 0.243 | 0.200 | 0.410 | 0.010 | 0.000 | 0.040 | 0.068 | 0.509 | 0.170 | 0.070 | 0.090 | 0.189 | 0.040 | 0.160 |
| Tibet | 0.000 | 0.000 | 0.000 | 0.000 | 0.000 | 0.003 | 0.100 | 0.270 | 0.000 | 0.000 | 0.000 | 0.000 | 0.034 | 0.000 | 0.000 | 0.000 | 0.000 | 0.000 | 0.040 |
| Shaanxi | 0.550 | 0.111 | 0.150 | 0.130 | 0.581 | 0.170 | 0.200 | 0.400 | 0.260 | 0.170 | 0.130 | 0.352 | 0.200 | 0.170 | 0.140 | 0.330 | 0.432 | 0.113 | 0.330 |
| Gansu | 0.160 | 0.072 | 0.050 | 0.060 | 0.210 | 0.135 | 0.000 | 0.180 | 0.010 | 0.010 | 0.040 | 0.176 | 0.339 | 0.000 | 0.000 | 0.060 | 0.148 | 0.025 | 0.160 |
| Qinghai | 0.000 | 0.011 | 0.010 | 0.010 | 0.078 | 0.027 | 0.000 | 0.490 | 0.010 | 0.000 | 0.000 | 0.100 | 0.001 | 0.170 | 0.000 | 0.040 | 0.007 | 0.002 | 0.250 |
| Ningxia | 0.000 | 0.011 | 0.010 | 0.020 | 0.072 | 0.050 | 0.000 | 0.240 | 0.100 | 0.020 | 0.020 | 0.134 | 0.057 | 0.000 | 0.000 | 0.020 | 0.043 | 0.006 | 0.090 |
| Xinjiang | 0.030 | 0.041 | 0.080 | 0.050 | 0.130 | 0.112 | 0.100 | 0.380 | 0.020 | 0.010 | 0.020 | 0.053 | 0.254 | 0.000 | 0.000 | 0.020 | 0.109 | 0.025 | 0.160 |

S1-4 CII Standardization Data in 2015

| **Scope of research** | **C1** | **C2** | **C3** | **C4** | **C5** | **C6** | **C7** | **C8** | **C9** | **C10** | **C11** | **C12** | **C13** | **C14** | **C15** | **C16** | **C17** | **C18** | **C19** |
| --- | --- | --- | --- | --- | --- | --- | --- | --- | --- | --- | --- | --- | --- | --- | --- | --- | --- | --- | --- |
| Beijing | 0.670 | 0.071 | 0.200 | 0.480 | 0.426 | 0.366 | 0.630 | 0.550 | 0.690 | 0.740 | 0.150 | 1.000 | 0.571 | 0.400 | 1.000 | 0.130 | 1.000 | 0.375 | 1.000 |
| Tianjin | 0.330 | 0.097 | 0.110 | 0.160 | 0.123 | 0.184 | 0.250 | 1.000 | 0.070 | 0.020 | 0.220 | 0.830 | 0.179 | 0.200 | 0.080 | 0.180 | 0.268 | 0.149 | 0.920 |
| Hebei | 0.270 | 0.164 | 0.120 | 0.150 | 0.447 | 0.265 | 0.130 | 0.480 | 0.400 | 0.220 | 0.160 | 0.195 | 0.179 | 0.000 | 0.080 | 0.160 | 0.250 | 0.120 | 0.510 |
| Shanxi | 0.060 | 0.094 | 0.070 | 0.090 | 0.329 | 0.242 | 0.000 | 0.580 | 0.080 | 0.070 | 0.040 | 0.155 | 0.264 | 0.000 | 0.040 | 0.080 | 0.124 | 0.039 | 0.230 |
| Inner Mongolia | 0.120 | 0.034 | 0.040 | 0.040 | 0.234 | 0.090 | 0.130 | 0.840 | 0.050 | 0.030 | 0.030 | 0.142 | 0.289 | 0.200 | 0.000 | 0.020 | 0.113 | 0.021 | 0.170 |
| Liaoning | 0.360 | 0.186 | 0.170 | 0.160 | 0.433 | 0.617 | 0.130 | 0.280 | 0.140 | 0.090 | 0.100 | 0.284 | 0.355 | 0.200 | 0.040 | 0.050 | 0.445 | 0.100 | 0.300 |
| Jilin | 0.210 | 0.077 | 0.060 | 0.090 | 0.264 | 0.241 | 0.130 | 0.390 | 0.070 | 0.020 | 0.040 | 0.210 | 0.449 | 0.000 | 0.040 | 0.190 | 0.284 | 0.035 | 0.080 |
| Heilongjiang | 0.240 | 0.056 | 0.040 | 0.040 | 0.321 | 0.164 | 0.130 | 0.550 | 0.010 | 0.010 | 0.060 | 0.198 | 0.670 | 0.400 | 0.080 | 0.030 | 0.315 | 0.075 | 0.000 |
| Shanghai | 0.300 | 0.138 | 0.140 | 0.190 | 0.259 | 0.299 | 1.000 | 0.170 | 0.380 | 0.430 | 0.210 | 0.601 | 0.469 | 1.000 | 0.640 | 0.370 | 0.655 | 0.242 | 0.750 |
| Jiangsu | 1.000 | 0.961 | 1.000 | 1.000 | 1.000 | 1.000 | 0.130 | 0.220 | 0.370 | 0.180 | 1.000 | 0.422 | 0.742 | 0.400 | 0.400 | 1.000 | 0.961 | 1.000 | 0.240 |
| Zhejiang | 0.300 | 1.000 | 0.790 | 0.560 | 0.605 | 0.760 | 0.380 | 0.020 | 0.380 | 0.170 | 1.000 | 0.382 | 0.451 | 0.000 | 0.080 | 0.330 | 0.358 | 0.939 | 0.270 |
| Anhui | 0.270 | 0.213 | 0.200 | 0.180 | 0.393 | 0.309 | 0.000 | 0.140 | 0.680 | 0.220 | 0.270 | 0.285 | 0.353 | 0.200 | 0.080 | 0.150 | 0.325 | 0.235 | 0.390 |
| Fujian | 0.360 | 0.374 | 0.380 | 0.260 | 0.386 | 0.440 | 0.130 | 0.100 | 0.050 | 0.030 | 0.210 | 0.221 | 0.338 | 0.200 | 0.080 | 0.150 | 0.187 | 0.246 | 0.110 |
| Jiangxi | 0.150 | 0.179 | 0.140 | 0.160 | 0.217 | 0.189 | 0.000 | 0.120 | 0.010 | 0.000 | 0.080 | 0.138 | 0.357 | 0.200 | 0.080 | 0.110 | 0.167 | 0.096 | 0.170 |
| Shandong | 0.420 | 0.381 | 0.340 | 0.410 | 0.741 | 0.661 | 0.380 | 0.300 | 0.460 | 0.330 | 0.590 | 0.431 | 1.000 | 0.400 | 0.320 | 0.250 | 0.447 | 0.391 | 0.250 |
| Henan | 0.300 | 0.329 | 0.270 | 0.340 | 0.439 | 0.569 | 0.250 | 0.480 | 0.390 | 0.330 | 0.230 | 0.164 | 0.276 | 0.600 | 0.240 | 0.160 | 0.424 | 0.190 | 0.200 |
| Hubei | 0.580 | 0.331 | 0.320 | 0.480 | 0.353 | 0.349 | 0.130 | 0.290 | 1.000 | 1.000 | 0.170 | 0.293 | 0.279 | 0.200 | 0.240 | 0.220 | 0.606 | 0.154 | 0.780 |
| Hunan | 0.450 | 0.265 | 0.210 | 0.210 | 0.325 | 0.219 | 0.000 | 0.350 | 0.560 | 0.360 | 0.130 | 0.217 | 0.610 | 0.200 | 0.120 | 0.200 | 0.402 | 0.135 | 0.250 |
| Guangdong | 0.550 | 0.278 | 0.320 | 0.390 | 0.697 | 0.544 | 0.250 | 0.410 | 0.310 | 0.220 | 0.720 | 0.399 | 0.840 | 0.600 | 0.200 | 0.460 | 0.593 | 0.964 | 0.570 |
| Guangxi | 0.090 | 0.119 | 0.090 | 0.050 | 0.228 | 0.103 | 0.000 | 0.020 | 0.020 | 0.150 | 0.460 | 0.079 | 0.546 | 0.000 | 0.160 | 0.160 | 0.209 | 0.053 | 0.240 |
| Hainan | 0.000 | 0.006 | 0.000 | 0.000 | 0.066 | 0.000 | 0.130 | 0.000 | 0.000 | 0.000 | 0.010 | 0.028 | 0.000 | 0.000 | 0.000 | 0.030 | 0.038 | 0.007 | 0.450 |
| Chongqing | 0.210 | 0.243 | 0.250 | 0.310 | 0.137 | 0.282 | 0.000 | 0.060 | 0.010 | 0.010 | 0.130 | 0.234 | 0.464 | 0.200 | 0.080 | 0.130 | 0.249 | 0.155 | 0.290 |
| Sichuan | 0.360 | 0.347 | 0.230 | 0.220 | 0.511 | 0.433 | 0.130 | 0.280 | 0.130 | 0.110 | 0.130 | 0.257 | 0.574 | 0.200 | 0.040 | 0.030 | 0.558 | 0.259 | 0.230 |
| Guizhou | 0.000 | 0.068 | 0.040 | 0.040 | 0.157 | 0.083 | 0.000 | 0.100 | 0.010 | 0.000 | 0.030 | 0.055 | 0.240 | 0.200 | 0.120 | 0.140 | 0.130 | 0.056 | 0.420 |
| Yunnan | 0.150 | 0.121 | 0.090 | 0.120 | 0.204 | 0.257 | 0.000 | 0.350 | 0.020 | 0.000 | 0.060 | 0.081 | 0.569 | 0.000 | 0.000 | 0.040 | 0.201 | 0.046 | 0.200 |
| Tibet | 0.000 | 0.000 | 0.000 | 0.000 | 0.000 | 0.000 | 0.000 | 0.590 | 0.000 | 0.000 | 0.000 | 0.000 | 0.045 | 0.000 | 0.000 | 0.000 | 0.000 | 0.000 | 0.270 |
| Shaanxi | 0.550 | 0.144 | 0.150 | 0.120 | 0.620 | 0.211 | 0.130 | 0.510 | 0.250 | 0.160 | 0.080 | 0.359 | 0.211 | 0.000 | 0.120 | 0.400 | 0.474 | 0.133 | 0.500 |
| Gansu | 0.150 | 0.069 | 0.050 | 0.060 | 0.239 | 0.138 | 0.000 | 0.360 | 0.010 | 0.000 | 0.030 | 0.181 | 0.384 | 0.400 | 0.000 | 0.080 | 0.139 | 0.027 | 0.260 |
| Qinghai | 0.000 | 0.010 | 0.010 | 0.010 | 0.087 | 0.031 | 0.000 | 0.680 | 0.000 | 0.000 | 0.000 | 0.053 | 0.005 | 0.000 | 0.040 | 0.010 | 0.010 | 0.004 | 0.310 |
| Ningxia | 0.000 | 0.013 | 0.010 | 0.010 | 0.083 | 0.047 | 0.000 | 0.250 | 0.100 | 0.020 | 0.020 | 0.130 | 0.054 | 0.000 | 0.040 | 0.000 | 0.033 | 0.007 | 0.130 |
| Xinjiang | 0.060 | 0.045 | 0.090 | 0.040 | 0.135 | 0.125 | 0.000 | 0.280 | 0.020 | 0.000 | 0.020 | 0.049 | 0.318 | 0.000 | 0.080 | 0.040 | 0.114 | 0.034 | 0.190 |

S1-5 CII Standardization Data in 2016

| **Scope of research** | **C1** | **C2** | **C3** | **C4** | **C5** | **C6** | **C7** | **C8** | **C9** | **C10** | **C11** | **C12** | **C13** | **C14** | **C15** | **C16** | **C17** | **C18** | **C19** |
| --- | --- | --- | --- | --- | --- | --- | --- | --- | --- | --- | --- | --- | --- | --- | --- | --- | --- | --- | --- |
| Beijing | 0.610 | 0.072 | 0.190 | 0.680 | 0.420 | 0.354 | 0.710 | 0.340 | 0.630 | 0.710 | 0.120 | 1.000 | 0.487 | 0.000 | 0.940 | 0.130 | 1.000 | 0.388 | 1.000 |
| Tianjin | 0.290 | 0.092 | 0.090 | 0.090 | 0.114 | 0.183 | 0.290 | 1.000 | 0.060 | 0.020 | 0.200 | 0.847 | 0.170 | 0.250 | 0.310 | 0.180 | 0.248 | 0.153 | 0.850 |
| Hebei | 0.290 | 0.167 | 0.120 | 0.150 | 0.417 | 0.283 | 0.290 | 0.220 | 0.370 | 0.220 | 0.160 | 0.219 | 0.157 | 0.000 | 0.130 | 0.160 | 0.301 | 0.122 | 0.450 |
| Shanxi | 0.050 | 0.095 | 0.070 | 0.090 | 0.311 | 0.275 | 0.000 | 0.390 | 0.070 | 0.060 | 0.040 | 0.174 | 0.237 | 0.250 | 0.060 | 0.080 | 0.150 | 0.038 | 0.140 |
| Inner Mongolia | 0.110 | 0.035 | 0.040 | 0.050 | 0.205 | 0.095 | 0.140 | 0.330 | 0.040 | 0.030 | 0.040 | 0.166 | 0.271 | 0.000 | 0.000 | 0.020 | 0.131 | 0.022 | 0.140 |
| Liaoning | 0.320 | 0.161 | 0.110 | 0.110 | 0.417 | 0.589 | 0.140 | 0.160 | 0.120 | 0.080 | 0.110 | 0.309 | 0.294 | 0.000 | 0.060 | 0.050 | 0.476 | 0.096 | 0.160 |
| Jilin | 0.180 | 0.071 | 0.050 | 0.080 | 0.237 | 0.235 | 0.140 | 0.480 | 0.070 | 0.020 | 0.040 | 0.217 | 0.354 | 0.000 | 0.060 | 0.190 | 0.312 | 0.038 | 0.100 |
| Heilongjiang | 0.210 | 0.045 | 0.040 | 0.040 | 0.276 | 0.162 | 0.140 | 0.410 | 0.010 | 0.010 | 0.050 | 0.206 | 0.559 | 1.000 | 0.130 | 0.030 | 0.300 | 0.069 | 0.000 |
| Shanghai | 0.260 | 0.132 | 0.130 | 0.210 | 0.235 | 0.290 | 1.000 | 0.170 | 0.360 | 0.400 | 0.180 | 0.626 | 0.400 | 1.000 | 1.000 | 0.370 | 0.670 | 0.247 | 0.800 |
| Jiangsu | 1.000 | 0.991 | 1.000 | 1.000 | 1.000 | 1.000 | 0.860 | 0.140 | 0.340 | 0.190 | 1.000 | 0.458 | 0.580 | 1.000 | 0.630 | 1.000 | 0.965 | 0.892 | 0.180 |
| Zhejiang | 0.260 | 1.000 | 0.760 | 0.570 | 0.611 | 0.774 | 0.290 | 0.030 | 0.350 | 0.170 | 0.990 | 0.415 | 0.416 | 0.250 | 0.130 | 0.330 | 0.415 | 0.855 | 0.240 |
| Anhui | 0.240 | 0.215 | 0.200 | 0.200 | 0.382 | 0.333 | 0.290 | 0.110 | 0.630 | 0.240 | 0.280 | 0.306 | 0.247 | 0.000 | 0.130 | 0.150 | 0.313 | 0.235 | 0.360 |
| Fujian | 0.390 | 0.420 | 0.370 | 0.270 | 0.432 | 0.471 | 0.000 | 0.060 | 0.050 | 0.030 | 0.220 | 0.253 | 0.338 | 0.000 | 0.130 | 0.160 | 0.210 | 0.259 | 0.030 |
| Jiangxi | 0.160 | 0.195 | 0.130 | 0.180 | 0.192 | 0.200 | 0.000 | 0.080 | 0.010 | 0.000 | 0.110 | 0.177 | 0.310 | 0.500 | 0.130 | 0.110 | 0.198 | 0.121 | 0.190 |
| Shandong | 0.390 | 0.378 | 0.350 | 0.410 | 0.727 | 0.679 | 0.290 | 0.190 | 0.420 | 0.330 | 0.600 | 0.468 | 1.000 | 0.250 | 0.500 | 0.250 | 0.451 | 0.378 | 0.200 |
| Henan | 0.340 | 0.336 | 0.360 | 0.440 | 0.403 | 0.644 | 0.290 | 0.210 | 0.350 | 0.340 | 0.210 | 0.196 | 0.275 | 0.250 | 0.380 | 0.160 | 0.413 | 0.189 | 0.240 |
| Hubei | 0.530 | 0.348 | 0.330 | 0.470 | 0.339 | 0.372 | 0.430 | 0.140 | 1.000 | 1.000 | 0.170 | 0.304 | 0.222 | 0.250 | 0.380 | 0.220 | 0.641 | 0.161 | 0.730 |
| Hunan | 0.420 | 0.283 | 0.210 | 0.220 | 0.313 | 0.227 | 0.290 | 0.330 | 0.520 | 0.370 | 0.130 | 0.251 | 0.433 | 0.000 | 0.190 | 0.200 | 0.419 | 0.131 | 0.230 |
| Guangdong | 0.470 | 0.294 | 0.330 | 0.410 | 0.701 | 0.568 | 0.430 | 0.270 | 0.290 | 0.220 | 0.850 | 0.432 | 0.855 | 0.250 | 0.310 | 0.480 | 0.737 | 1.000 | 0.590 |
| Guangxi | 0.080 | 0.153 | 0.090 | 0.060 | 0.211 | 0.113 | 0.290 | 0.000 | 0.020 | 0.140 | 0.040 | 0.102 | 0.443 | 0.250 | 0.250 | 0.160 | 0.194 | 0.056 | 0.170 |
| Hainan | 0.000 | 0.006 | 0.000 | 0.000 | 0.038 | 0.000 | 0.290 | 0.030 | 0.000 | 0.000 | 0.010 | 0.064 | 0.041 | 0.250 | 0.060 | 0.030 | 0.034 | 0.007 | 0.440 |
| Chongqing | 0.210 | 0.269 | 0.250 | 0.320 | 0.117 | 0.298 | 0.290 | 0.020 | 0.010 | 0.010 | 0.130 | 0.281 | 0.484 | 0.250 | 0.130 | 0.130 | 0.267 | 0.164 | 0.240 |
| Sichuan | 0.340 | 0.365 | 0.220 | 0.260 | 0.466 | 0.484 | 0.140 | 0.080 | 0.120 | 0.110 | 0.150 | 0.283 | 0.479 | 0.000 | 0.060 | 0.030 | 0.559 | 0.240 | 0.180 |
| Guizhou | 0.000 | 0.084 | 0.050 | 0.050 | 0.130 | 0.098 | 0.140 | 0.110 | 0.010 | 0.010 | 0.040 | 0.081 | 0.223 | 0.500 | 0.190 | 0.140 | 0.137 | 0.039 | 0.270 |
| Yunnan | 0.130 | 0.147 | 0.100 | 0.140 | 0.217 | 0.276 | 0.140 | 0.170 | 0.020 | 0.010 | 0.060 | 0.117 | 0.514 | 0.000 | 0.000 | 0.040 | 0.207 | 0.046 | 0.130 |
| Tibet | 0.000 | 0.000 | 0.000 | 0.000 | 0.000 | 0.003 | 0.000 | 0.340 | 0.000 | 0.000 | 0.000 | 0.000 | 0.033 | 0.000 | 0.000 | 0.000 | 0.000 | 0.000 | 0.280 |
| Shaanxi | 0.470 | 0.150 | 0.160 | 0.150 | 0.612 | 0.226 | 0.140 | 0.230 | 0.220 | 0.160 | 0.070 | 0.379 | 0.185 | 0.000 | 0.190 | 0.400 | 0.505 | 0.186 | 0.480 |
| Gansu | 0.180 | 0.070 | 0.050 | 0.050 | 0.229 | 0.142 | 0.140 | 0.210 | 0.010 | 0.000 | 0.020 | 0.202 | 0.317 | 0.250 | 0.000 | 0.080 | 0.150 | 0.030 | 0.190 |
| Qinghai | 0.000 | 0.011 | 0.010 | 0.000 | 0.059 | 0.028 | 0.000 | 0.390 | 0.000 | 0.000 | 0.000 | 0.081 | 0.000 | 0.000 | 0.060 | 0.010 | 0.014 | 0.004 | 0.100 |
| Ningxia | 0.000 | 0.009 | 0.010 | 0.010 | 0.050 | 0.051 | 0.000 | 0.200 | 0.100 | 0.020 | 0.020 | 0.168 | 0.049 | 0.250 | 0.060 | 0.000 | 0.038 | 0.009 | 0.040 |
| Xinjiang | 0.050 | 0.046 | 0.080 | 0.040 | 0.125 | 0.130 | 0.000 | 0.230 | 0.020 | 0.000 | 0.020 | 0.075 | 0.270 | 0.250 | 0.130 | 0.010 | 0.113 | 0.027 | 0.140 |

S1-6 CII Standardization Data in 2017

| **Scope of research** | **C1** | **C2** | **C3** | **C4** | **C5** | **C6** | **C7** | **C8** | **C9** | **C10** | **C11** | **C12** | **C13** | **C14** | **C15** | **C16** | **C17** | **C18** | **C19** |
| --- | --- | --- | --- | --- | --- | --- | --- | --- | --- | --- | --- | --- | --- | --- | --- | --- | --- | --- | --- |
| Beijing | 0.62 | 0.07 | 0.19 | 0.68 | 0.41 | 0.32 | 0.78 | 0.48 | 0.66 | 0.71 | 0.11 | 1.00 | 0.55 | 0.00 | 1.00 | 0.07 | 1.00 | 0.32 | 1.00 |
| Tianjin | 0.28 | 0.07 | 0.07 | 0.09 | 0.09 | 0.20 | 0.11 | 1.00 | 0.04 | 0.01 | 0.18 | 0.68 | 0.15 | 0.20 | 0.31 | 0.06 | 0.22 | 0.12 | 0.65 |
| Hebei | 0.28 | 0.17 | 0.11 | 0.15 | 0.38 | 0.29 | 0.00 | 0.41 | 0.36 | 0.24 | 0.15 | 0.25 | 0.18 | 0.00 | 0.13 | 0.16 | 0.29 | 0.11 | 0.41 |
| Shanxi | 0.08 | 0.10 | 0.07 | 0.09 | 0.26 | 0.28 | 0.00 | 0.52 | 0.08 | 0.07 | 0.05 | 0.16 | 0.26 | 0.20 | 0.06 | 0.08 | 0.15 | 0.03 | 0.31 |
| Inner Mongolia | 0.10 | 0.03 | 0.03 | 0.05 | 0.17 | 0.10 | 0.00 | 0.51 | 0.03 | 0.02 | 0.03 | 0.13 | 0.27 | 0.00 | 0.00 | 0.02 | 0.11 | 0.02 | 0.22 |
| Liaoning | 0.33 | 0.13 | 0.09 | 0.11 | 0.39 | 0.59 | 0.22 | 0.26 | 0.13 | 0.09 | 0.12 | 0.35 | 0.34 | 0.00 | 0.06 | 0.05 | 0.42 | 0.08 | 0.27 |
| Jilin | 0.18 | 0.06 | 0.04 | 0.08 | 0.21 | 0.25 | 0.00 | 0.26 | 0.06 | 0.01 | 0.03 | 0.19 | 0.37 | 0.00 | 0.06 | 0.19 | 0.26 | 0.03 | 0.35 |
| Heilongjiang | 0.23 | 0.04 | 0.03 | 0.04 | 0.25 | 0.17 | 0.00 | 0.62 | 0.01 | 0.01 | 0.05 | 0.19 | 0.60 | 0.20 | 0.13 | 0.03 | 0.29 | 0.05 | 0.05 |
| Shanghai | 0.26 | 0.11 | 0.12 | 0.21 | 0.22 | 0.31 | 1.00 | 0.29 | 0.33 | 0.42 | 0.17 | 0.68 | 0.47 | 1.00 | 1.00 | 0.37 | 0.66 | 0.22 | 0.90 |
| Jiangsu | 1.00 | 0.97 | 1.00 | 1.00 | 1.00 | 1.00 | 0.44 | 0.19 | 0.35 | 0.19 | 0.92 | 0.48 | 0.72 | 0.40 | 0.63 | 1.00 | 0.96 | 0.68 | 0.22 |
| Zhejiang | 0.28 | 1.00 | 0.74 | 0.57 | 0.65 | 0.72 | 0.44 | 0.05 | 0.37 | 0.18 | 0.94 | 0.44 | 0.47 | 0.20 | 0.13 | 0.33 | 0.41 | 0.64 | 0.32 |
| Anhui | 0.31 | 0.21 | 0.19 | 0.20 | 0.37 | 0.36 | 0.11 | 0.17 | 0.67 | 0.26 | 0.27 | 0.33 | 0.28 | 0.20 | 0.13 | 0.15 | 0.30 | 0.17 | 0.45 |
| Fujian | 0.44 | 0.48 | 0.41 | 0.27 | 0.48 | 0.53 | 0.11 | 0.04 | 0.05 | 0.03 | 0.22 | 0.27 | 0.37 | 0.20 | 0.13 | 0.15 | 0.22 | 0.20 | 0.06 |
| Jiangxi | 0.15 | 0.20 | 0.13 | 0.18 | 0.17 | 0.26 | 0.00 | 0.15 | 0.02 | 0.00 | 0.10 | 0.21 | 0.36 | 0.00 | 0.13 | 0.11 | 0.18 | 0.10 | 0.08 |
| Shandong | 0.38 | 0.41 | 0.35 | 0.41 | 0.72 | 0.77 | 0.11 | 0.28 | 0.42 | 0.35 | 0.59 | 0.51 | 0.98 | 0.00 | 0.50 | 0.25 | 0.48 | 0.30 | 0.27 |
| Henan | 0.36 | 0.34 | 0.33 | 0.44 | 0.36 | 0.73 | 0.11 | 0.29 | 0.34 | 0.37 | 0.22 | 0.21 | 0.33 | 0.00 | 0.38 | 0.16 | 0.36 | 0.17 | 0.35 |
| Hubei | 0.51 | 0.36 | 0.31 | 0.47 | 0.33 | 0.42 | 0.22 | 0.18 | 1.00 | 1.00 | 0.18 | 0.33 | 0.23 | 0.00 | 0.38 | 0.22 | 0.60 | 0.14 | 0.88 |
| Hunan | 0.46 | 0.31 | 0.20 | 0.22 | 0.30 | 0.26 | 0.56 | 0.26 | 0.58 | 0.41 | 0.14 | 0.29 | 0.44 | 0.60 | 0.19 | 0.20 | 0.43 | 0.11 | 0.22 |
| Guangdong | 0.46 | 0.33 | 0.34 | 0.41 | 0.72 | 0.64 | 0.22 | 0.24 | 0.32 | 0.23 | 1.00 | 0.46 | 1.00 | 0.60 | 0.31 | 0.46 | 0.69 | 1.00 | 0.59 |
| Guangxi | 0.10 | 0.16 | 0.09 | 0.06 | 0.20 | 0.13 | 0.11 | 0.00 | 0.02 | 0.16 | 0.04 | 0.12 | 0.46 | 0.20 | 0.25 | 0.12 | 0.17 | 0.04 | 0.18 |
| Hainan | 0.00 | 0.00 | 0.00 | 0.00 | 0.02 | 0.00 | 0.11 | 0.05 | 0.00 | 0.00 | 0.01 | 0.06 | 0.01 | 0.40 | 0.06 | 0.03 | 0.03 | 0.01 | 0.48 |
| Chongqing | 0.21 | 0.28 | 0.24 | 0.32 | 0.09 | 0.32 | 0.00 | 0.01 | 0.01 | 0.01 | 0.14 | 0.32 | 0.49 | 0.20 | 0.13 | 0.13 | 0.25 | 0.10 | 0.23 |
| Sichuan | 0.33 | 0.44 | 0.22 | 0.26 | 0.44 | 0.59 | 0.11 | 0.12 | 0.15 | 0.12 | 0.17 | 0.29 | 0.53 | 0.40 | 0.06 | 0.03 | 0.53 | 0.19 | 0.14 |
| Guizhou | 0.00 | 0.09 | 0.06 | 0.05 | 0.15 | 0.10 | 0.22 | 0.17 | 0.02 | 0.01 | 0.04 | 0.10 | 0.25 | 0.00 | 0.19 | 0.14 | 0.13 | 0.04 | 0.38 |
| Yunnan | 0.13 | 0.19 | 0.10 | 0.14 | 0.19 | 0.29 | 0.00 | 0.13 | 0.02 | 0.01 | 0.06 | 0.13 | 0.58 | 0.20 | 0.00 | 0.04 | 0.16 | 0.04 | 0.22 |
| Tibet | 0.00 | 0.00 | 0.00 | 0.00 | 0.00 | 0.01 | 0.00 | 0.46 | 0.00 | 0.00 | 0.00 | 0.00 | 0.02 | 0.00 | 0.00 | 0.00 | 0.00 | 0.00 | 0.22 |
| Shaanxi | 0.49 | 0.17 | 0.16 | 0.15 | 0.62 | 0.26 | 0.00 | 0.23 | 0.23 | 0.16 | 0.07 | 0.38 | 0.20 | 0.20 | 0.19 | 0.40 | 0.49 | 0.10 | 0.51 |
| Gansu | 0.18 | 0.07 | 0.04 | 0.05 | 0.21 | 0.15 | 0.11 | 0.29 | 0.01 | 0.00 | 0.02 | 0.19 | 0.33 | 0.20 | 0.00 | 0.08 | 0.14 | 0.03 | 0.16 |
| Qinghai | 0.00 | 0.01 | 0.01 | 0.00 | 0.03 | 0.03 | 0.00 | 0.94 | 0.00 | 0.00 | 0.00 | 0.10 | 0.00 | 0.00 | 0.06 | 0.01 | 0.01 | 0.00 | 0.13 |
| Ningxia | 0.00 | 0.01 | 0.01 | 0.01 | 0.03 | 0.07 | 0.00 | 0.17 | 0.11 | 0.03 | 0.02 | 0.20 | 0.04 | 0.00 | 0.06 | 0.00 | 0.04 | 0.01 | 0.00 |
| Xinjiang | 0.05 | 0.05 | 0.07 | 0.04 | 0.11 | 0.13 | 0.00 | 0.21 | 0.01 | 0.00 | 0.02 | 0.06 | 0.27 | 0.00 | 0.13 | 0.02 | 0.10 | 0.02 | 0.21 |

S1-7 CII Standardization Data in 2018

| **Scope of research** | **C1** | **C2** | **C3** | **C4** | **C5** | **C6** | **C7** | **C8** | **C9** | **C10** | **C11** | **C12** | **C13** | **C14** | **C15** | **C16** | **C17** | **C18** | **C19** |
| --- | --- | --- | --- | --- | --- | --- | --- | --- | --- | --- | --- | --- | --- | --- | --- | --- | --- | --- | --- |
| Beijing | 0.60 | 0.06 | 0.17 | 0.42 | 0.43 | 0.29 | 0.75 | 0.30 | 0.53 | 0.65 | 0.09 | 1.00 | 0.58 | 0.17 | 1.00 | 0.07 | 0.98 | 0.26 | 0.87 |
| Tianjin | 0.28 | 0.07 | 0.06 | 0.07 | 0.15 | 0.20 | 0.13 | 0.41 | 0.04 | 0.01 | 0.15 | 0.64 | 0.17 | 0.17 | 0.31 | 0.06 | 0.26 | 0.11 | 0.40 |
| Hebei | 0.28 | 0.15 | 0.12 | 0.11 | 0.42 | 0.25 | 0.00 | 1.00 | 0.29 | 0.23 | 0.13 | 0.24 | 0.18 | 0.17 | 0.13 | 0.16 | 0.25 | 0.11 | 0.50 |
| Shanxi | 0.08 | 0.09 | 0.07 | 0.07 | 0.30 | 0.27 | 0.00 | 0.25 | 0.06 | 0.07 | 0.04 | 0.16 | 0.26 | 0.00 | 0.06 | 0.08 | 0.16 | 0.03 | 0.33 |
| Inner Mongolia | 0.10 | 0.03 | 0.03 | 0.03 | 0.21 | 0.10 | 0.13 | 0.25 | 0.02 | 0.02 | 0.03 | 0.10 | 0.27 | 0.17 | 0.00 | 0.02 | 0.09 | 0.02 | 0.16 |
| Liaoning | 0.33 | 0.09 | 0.09 | 0.09 | 0.38 | 0.54 | 0.13 | 0.15 | 0.12 | 0.09 | 0.12 | 0.32 | 0.06 | 0.00 | 0.06 | 0.05 | 0.41 | 0.07 | 0.28 |
| Jilin | 0.18 | 0.04 | 0.05 | 0.07 | 0.24 | 0.23 | 0.00 | 0.16 | 0.03 | 0.01 | 0.02 | 0.14 | 0.35 | 0.00 | 0.06 | 0.19 | 0.26 | 0.03 | 0.40 |
| Heilongjiang | 0.28 | 0.03 | 0.03 | 0.01 | 0.28 | 0.17 | 0.00 | 0.32 | 0.00 | 0.00 | 0.03 | 0.15 | 0.56 | 0.17 | 0.13 | 0.03 | 0.31 | 0.04 | 0.00 |
| Shanghai | 0.28 | 0.10 | 0.13 | 0.19 | 0.28 | 0.25 | 1.00 | 0.13 | 0.29 | 0.39 | 0.16 | 0.65 | 0.45 | 0.33 | 1.00 | 0.37 | 0.67 | 0.19 | 0.97 |
| Jiangsu | 1.00 | 1.00 | 1.00 | 1.00 | 1.00 | 1.00 | 0.25 | 0.09 | 0.31 | 0.19 | 0.93 | 0.45 | 0.69 | 1.00 | 0.63 | 1.00 | 1.00 | 0.64 | 0.22 |
| Zhejiang | 0.30 | 0.98 | 0.72 | 0.42 | 0.69 | 0.72 | 0.25 | 0.01 | 0.39 | 0.17 | 1.00 | 0.42 | 0.46 | 0.17 | 0.13 | 0.33 | 0.41 | 0.59 | 0.29 |
| Anhui | 0.33 | 0.23 | 0.19 | 0.21 | 0.45 | 0.41 | 0.13 | 0.07 | 0.62 | 0.27 | 0.21 | 0.31 | 0.26 | 0.17 | 0.13 | 0.15 | 0.29 | 0.17 | 0.48 |
| Fujian | 0.48 | 0.53 | 0.39 | 0.33 | 0.59 | 0.59 | 0.38 | 0.02 | 0.05 | 0.04 | 0.24 | 0.26 | 0.35 | 0.00 | 0.13 | 0.15 | 0.21 | 0.21 | 0.01 |
| Jiangxi | 0.18 | 0.19 | 0.17 | 0.19 | 0.23 | 0.27 | 0.13 | 0.07 | 0.02 | 0.00 | 0.17 | 0.21 | 0.36 | 0.17 | 0.13 | 0.11 | 0.18 | 0.11 | 0.27 |
| Shandong | 0.38 | 0.40 | 0.37 | 0.34 | 0.73 | 0.77 | 0.13 | 0.11 | 0.38 | 0.28 | 0.60 | 0.41 | 1.00 | 0.17 | 0.50 | 0.25 | 0.53 | 0.28 | 0.31 |
| Henan | 0.43 | 0.36 | 0.32 | 0.46 | 0.41 | 0.72 | 0.00 | 0.13 | 0.31 | 0.37 | 0.21 | 0.20 | 0.36 | 0.17 | 0.38 | 0.16 | 0.37 | 0.17 | 0.33 |
| Hubei | 0.53 | 0.30 | 0.33 | 0.63 | 0.35 | 0.44 | 0.13 | 0.15 | 1.00 | 1.00 | 0.17 | 0.32 | 0.25 | 0.17 | 0.38 | 0.22 | 0.61 | 0.13 | 1.00 |
| Hunan | 0.48 | 0.32 | 0.21 | 0.27 | 0.35 | 0.27 | 0.25 | 0.12 | 0.57 | 0.41 | 0.20 | 0.29 | 0.41 | 0.00 | 0.19 | 0.20 | 0.44 | 0.10 | 0.25 |
| Guangdong | 0.50 | 0.34 | 0.35 | 0.46 | 0.74 | 0.69 | 1.00 | 0.14 | 0.39 | 0.24 | 0.99 | 0.46 | 0.92 | 0.83 | 0.31 | 0.46 | 0.78 | 1.00 | 0.62 |
| Guangxi | 0.10 | 0.16 | 0.09 | 0.07 | 0.27 | 0.13 | 0.25 | 0.00 | 0.02 | 0.13 | 0.04 | 0.09 | 0.48 | 0.17 | 0.25 | 0.12 | 0.17 | 0.04 | 0.20 |
| Hainan | 0.00 | 0.00 | 0.00 | 0.00 | 0.07 | 0.00 | 0.00 | 0.02 | 0.00 | 0.00 | 0.01 | 0.06 | 0.00 | 0.00 | 0.06 | 0.03 | 0.03 | 0.01 | 0.40 |
| Chongqing | 0.23 | 0.28 | 0.23 | 0.25 | 0.15 | 0.30 | 0.13 | 0.00 | 0.01 | 0.01 | 0.16 | 0.31 | 0.57 | 0.17 | 0.13 | 0.13 | 0.25 | 0.09 | 0.19 |
| Sichuan | 0.35 | 0.43 | 0.23 | 0.41 | 0.55 | 0.62 | 0.25 | 0.04 | 0.15 | 0.13 | 0.15 | 0.27 | 0.48 | 0.67 | 0.06 | 0.03 | 0.57 | 0.18 | 0.16 |
| Guizhou | 0.03 | 0.10 | 0.06 | 0.10 | 0.24 | 0.11 | 0.00 | 0.07 | 0.02 | 0.01 | 0.04 | 0.10 | 0.26 | 0.00 | 0.19 | 0.14 | 0.12 | 0.04 | 0.30 |
| Yunnan | 0.15 | 0.15 | 0.09 | 0.20 | 0.22 | 0.29 | 0.13 | 0.53 | 0.02 | 0.01 | 0.05 | 0.12 | 0.54 | 0.33 | 0.00 | 0.04 | 0.17 | 0.04 | 0.15 |
| Tibet | 0.00 | 0.00 | 0.00 | 0.01 | 0.00 | 0.01 | 0.00 | 0.10 | 0.00 | 0.00 | 0.00 | 0.00 | 0.02 | 0.17 | 0.00 | 0.00 | 0.00 | 0.00 | 0.10 |
| Shaanxi | 0.48 | 0.19 | 0.15 | 0.18 | 0.56 | 0.27 | 0.25 | 0.10 | 0.18 | 0.16 | 0.06 | 0.37 | 0.18 | 0.00 | 0.19 | 0.40 | 0.54 | 0.09 | 0.44 |
| Gansu | 0.18 | 0.06 | 0.04 | 0.04 | 0.25 | 0.15 | 0.00 | 0.14 | 0.01 | 0.00 | 0.02 | 0.18 | 0.30 | 0.00 | 0.00 | 0.08 | 0.12 | 0.03 | 0.17 |
| Qinghai | 0.00 | 0.01 | 0.01 | 0.00 | 0.09 | 0.02 | 0.00 | 0.27 | 0.00 | 0.00 | 0.00 | 0.07 | 0.00 | 0.00 | 0.06 | 0.01 | 0.01 | 0.00 | 0.34 |
| Ningxia | 0.00 | 0.01 | 0.01 | 0.00 | 0.08 | 0.06 | 0.00 | 0.08 | 0.11 | 0.03 | 0.02 | 0.20 | 0.03 | 0.00 | 0.06 | 0.00 | 0.03 | 0.01 | 0.01 |
| Xinjiang | 0.05 | 0.04 | 0.07 | 0.03 | 0.16 | 0.12 | 0.00 | 0.10 | 0.01 | 0.00 | 0.01 | 0.05 | 0.25 | 0.17 | 0.13 | 0.02 | 0.09 | 0.02 | 0.16 |

S1-8 CII Standardization Data in 2019

| **Scope of research** | **C1** | **C2** | **C3** | **C4** | **C5** | **C6** | **C7** | **C8** | **C9** | **C10** | **C11** | **C12** | **C13** | **C14** | **C15** | **C16** | **C17** | **C18** | **C19** |
| --- | --- | --- | --- | --- | --- | --- | --- | --- | --- | --- | --- | --- | --- | --- | --- | --- | --- | --- | --- |
| Beijing | 0.714 | 0.063 | 0.193 | 0.632 | 0.393 | 0.291 | 1.000 | 0.409 | 0.390 | 0.720 | 1.000 | 1.000 | 0.425 | 0.200 | 0.800 | 0.402 | 0.928 | 0.248 | 0.754 |
| Tianjin | 0.321 | 0.096 | 0.063 | 0.073 | 0.104 | 0.191 | 0.235 | 0.109 | 0.113 | 0.148 | 0.232 | 0.502 | 0.126 | 0.000 | 0.400 | 0.393 | 0.247 | 0.108 | 0.375 |
| Hebei | 0.393 | 0.106 | 0.300 | 0.107 | 0.356 | 0.271 | 0.000 | 0.140 | 0.137 | 0.182 | 0.243 | 0.225 | 0.146 | 0.000 | 0.400 | 0.094 | 0.244 | 0.108 | 0.821 |
| Shanxi | 0.107 | 0.096 | 0.096 | 0.067 | 0.242 | 0.304 | 0.000 | 0.109 | 0.056 | 0.060 | 0.142 | 0.144 | 0.175 | 0.200 | 0.200 | 0.017 | 0.158 | 0.030 | 0.350 |
| Inner Mongolia | 0.071 | 0.020 | 0.164 | 0.020 | 0.134 | 0.106 | 0.000 | 0.168 | 0.029 | 0.046 | 0.090 | 0.099 | 0.195 | 0.000 | 0.400 | 0.051 | 0.085 | 0.019 | 0.337 |
| Liaoning | 0.393 | 0.078 | 0.194 | 0.061 | 0.331 | 0.559 | 0.000 | 1.000 | 0.122 | 0.163 | 0.339 | 0.296 | 0.113 | 0.000 | 0.300 | 0.128 | 0.386 | 0.074 | 0.334 |
| Jilin | 0.286 | 0.042 | 0.082 | 0.059 | 0.207 | 0.237 | 0.118 | 0.081 | 0.051 | 0.047 | 0.240 | 0.167 | 0.258 | 0.000 | 0.100 | 0.017 | 0.247 | 0.028 | 0.420 |
| Heilongjiang | 0.250 | 0.028 | 0.017 | 0.012 | 0.203 | 0.178 | 0.000 | 0.148 | 0.053 | 0.046 | 0.188 | 0.136 | 0.410 | 0.200 | 0.100 | 0.060 | 0.314 | 0.036 | 0.084 |
| Shanghai | 0.286 | 0.102 | 0.067 | 0.173 | 0.249 | 0.243 | 0.765 | 0.138 | 0.246 | 0.491 | 0.596 | 0.621 | 0.369 | 1.000 | 0.300 | 1.000 | 0.691 | 0.189 | 0.859 |
| Jiangsu | 1.000 | 1.000 | 1.000 | 1.000 | 1.000 | 1.000 | 0.176 | 0.052 | 0.790 | 0.897 | 0.776 | 0.424 | 0.483 | 0.400 | 1.000 | 0.615 | 1.000 | 0.595 | 0.230 |
| Zhejiang | 0.250 | 0.750 | 0.585 | 0.352 | 0.713 | 0.771 | 0.059 | 0.023 | 0.665 | 0.538 | 0.684 | 0.399 | 0.348 | 0.200 | 1.000 | 0.222 | 0.404 | 0.540 | 0.146 |
| Anhui | 0.393 | 0.243 | 0.581 | 0.182 | 0.532 | 0.477 | 0.059 | 0.035 | 0.217 | 0.242 | 0.306 | 0.296 | 0.187 | 0.200 | 0.400 | 0.171 | 0.286 | 0.155 | 0.390 |
| Fujian | 0.500 | 0.568 | 0.693 | 0.310 | 0.635 | 0.642 | 0.000 | 0.021 | 0.212 | 0.242 | 0.371 | 0.252 | 0.283 | 0.200 | 0.300 | 0.162 | 0.204 | 0.186 | 0.000 |
| Jiangxi | 0.143 | 0.202 | 0.289 | 0.184 | 0.193 | 0.315 | 0.059 | 0.036 | 0.130 | 0.123 | 0.224 | 0.215 | 0.277 | 0.000 | 0.400 | 0.043 | 0.175 | 0.110 | 0.299 |
| Shandong | 0.393 | 0.386 | 0.867 | 0.315 | 0.746 | 0.776 | 0.000 | 0.106 | 0.346 | 0.482 | 0.520 | 0.308 | 0.638 | 0.000 | 0.900 | 0.291 | 0.530 | 0.276 | 0.355 |
| Henan | 0.500 | 0.367 | 0.808 | 0.453 | 0.427 | 0.776 | 0.118 | 0.063 | 0.237 | 0.255 | 0.292 | 0.202 | 0.259 | 0.000 | 0.300 | 0.094 | 0.350 | 0.162 | 0.332 |
| Hubei | 0.536 | 0.301 | 0.458 | 0.546 | 0.333 | 0.476 | 0.176 | 0.077 | 0.220 | 0.308 | 0.489 | 0.306 | 0.200 | 0.000 | 0.400 | 0.154 | 0.563 | 0.139 | 1.000 |
| Hunan | 0.500 | 0.342 | 0.516 | 0.261 | 0.316 | 0.303 | 0.000 | 0.033 | 0.194 | 0.253 | 0.441 | 0.283 | 0.300 | 0.600 | 0.400 | 0.051 | 0.432 | 0.102 | 0.239 |
| Guangdong | 0.536 | 0.407 | 0.668 | 0.445 | 0.702 | 0.762 | 0.118 | 0.080 | 1.000 | 1.000 | 0.788 | 0.432 | 1.000 | 0.800 | 0.800 | 0.487 | 0.750 | 1.000 | 0.509 |
| Guangxi | 0.107 | 0.172 | 0.249 | 0.075 | 0.252 | 0.154 | 0.294 | 0.000 | 0.057 | 0.053 | 0.224 | 0.088 | 0.343 | 0.200 | 0.400 | 0.068 | 0.170 | 0.041 | 0.269 |
| Hainan | 0.000 | 0.004 | 0.029 | 0.008 | 0.027 | 0.000 | 0.000 | 0.016 | 0.009 | 0.008 | 0.035 | 0.050 | 0.007 | 0.400 | 0.200 | 0.026 | 0.031 | 0.006 | 0.424 |
| Chongqing | 0.250 | 0.266 | 0.420 | 0.240 | 0.120 | 0.322 | 0.118 | 0.006 | 0.120 | 0.150 | 0.277 | 0.286 | 0.425 | 0.200 | 0.500 | 0.060 | 0.264 | 0.081 | 0.194 |
| Sichuan | 0.393 | 0.435 | 0.637 | 0.319 | 0.604 | 0.638 | 0.118 | 0.034 | 0.211 | 0.280 | 0.576 | 0.268 | 0.363 | 0.400 | 0.300 | 0.179 | 0.535 | 0.154 | 0.203 |
| Guizhou | 0.071 | 0.095 | 0.200 | 0.076 | 0.184 | 0.134 | 0.118 | 0.034 | 0.045 | 0.045 | 0.152 | 0.099 | 0.207 | 0.000 | 0.200 | 0.068 | 0.133 | 0.045 | 0.343 |
| Yunnan | 0.179 | 0.172 | 0.390 | 0.199 | 0.177 | 0.321 | 0.118 | 0.040 | 0.069 | 0.070 | 0.160 | 0.114 | 0.373 | 0.200 | 0.400 | 0.068 | 0.166 | 0.040 | 0.167 |
| Tibet | 0.000 | 0.000 | 0.031 | 0.020 | 0.000 | 0.006 | 0.000 | 0.029 | 0.000 | 0.000 | 0.002 | 0.000 | 0.023 | 0.000 | 0.000 | 0.000 | 0.000 | 0.000 | 0.145 |
| Shaanxi | 0.536 | 0.176 | 0.352 | 0.145 | 0.488 | 0.312 | 0.118 | 0.060 | 0.142 | 0.188 | 0.495 | 0.333 | 0.131 | 0.200 | 0.700 | 0.068 | 0.542 | 0.082 | 0.471 |
| Gansu | 0.143 | 0.058 | 0.039 | 0.051 | 0.222 | 0.175 | 0.000 | 0.078 | 0.030 | 0.034 | 0.101 | 0.166 | 0.215 | 0.000 | 0.100 | 0.051 | 0.118 | 0.026 | 0.208 |
| Qinghai | 0.000 | 0.004 | 0.003 | 0.000 | 0.043 | 0.027 | 0.000 | 0.161 | 0.005 | 0.005 | 0.000 | 0.073 | 0.000 | 0.000 | 0.100 | 0.000 | 0.013 | 0.004 | 0.418 |
| Ningxia | 0.000 | 0.008 | 0.000 | 0.007 | 0.029 | 0.057 | 0.000 | 0.035 | 0.013 | 0.016 | 0.033 | 0.197 | 0.025 | 0.000 | 0.100 | 0.026 | 0.034 | 0.009 | 0.060 |
| Xinjiang | 0.036 | 0.044 | 0.121 | 0.028 | 0.140 | 0.134 | 0.059 | 0.065 | 0.015 | 0.019 | 0.068 | 0.035 | 0.165 | 0.000 | 0.100 | 0.017 | 0.075 | 0.014 | 0.234 |

S1-9 CII Standardization Data in 2020

| **Scope of research** | **C1** | **C2** | **C3** | **C4** | **C5** | **C6** | **C7** | **C8** | **C9** | **C10** | **C11** | **C12** | **C13** | **C14** | **C15** | **C16** | **C17** | **C18** | **C19** |
| --- | --- | --- | --- | --- | --- | --- | --- | --- | --- | --- | --- | --- | --- | --- | --- | --- | --- | --- | --- |
| Beijing | 0.645 | 0.055 | 0.197 | 0.764 | 0.341 | 0.209 | 1.000 | 1.000 | 0.384 | 0.668 | 1.000 | 1.000 | 0.400 | 0.200 | 0.750 | 0.402 | 0.862 | 0.228 | 0.683 |
| Tianjin | 0.258 | 0.071 | 0.064 | 0.038 | 0.070 | 0.181 | 0.059 | 0.204 | 0.102 | 0.138 | 0.232 | 0.517 | 0.118 | 0.200 | 0.250 | 0.393 | 0.229 | 0.104 | 0.229 |
| Hebei | 0.484 | 0.090 | 0.286 | 0.085 | 0.321 | 0.250 | 0.000 | 0.282 | 0.142 | 0.181 | 0.253 | 0.245 | 0.140 | 0.200 | 0.500 | 0.094 | 0.232 | 0.128 | 0.682 |
| Shanxi | 0.129 | 0.097 | 0.102 | 0.077 | 0.200 | 0.288 | 0.176 | 0.238 | 0.058 | 0.059 | 0.153 | 0.156 | 0.161 | 0.000 | 0.250 | 0.017 | 0.172 | 0.036 | 0.290 |
| Inner Mongolia | 0.129 | 0.015 | 0.162 | 0.008 | 0.070 | 0.085 | 0.118 | 0.391 | 0.030 | 0.045 | 0.085 | 0.113 | 0.181 | 0.000 | 0.167 | 0.051 | 0.086 | 0.023 | 0.401 |
| Liaoning | 0.419 | 0.065 | 0.196 | 0.039 | 0.289 | 0.500 | 0.059 | 0.339 | 0.127 | 0.157 | 0.322 | 0.316 | 0.083 | 0.000 | 0.083 | 0.128 | 0.389 | 0.083 | 0.420 |
| Jilin | 0.258 | 0.031 | 0.083 | 0.041 | 0.161 | 0.210 | 0.000 | 0.248 | 0.049 | 0.045 | 0.258 | 0.172 | 0.231 | 0.000 | 0.083 | 0.017 | 0.247 | 0.031 | 0.529 |
| Heilongjiang | 0.194 | 0.021 | 0.014 | 0.005 | 0.161 | 0.184 | 0.059 | 0.341 | 0.049 | 0.049 | 0.184 | 0.166 | 0.372 | 0.000 | 0.167 | 0.060 | 0.291 | 0.038 | 0.119 |
| Shanghai | 0.258 | 0.086 | 0.063 | 0.141 | 0.221 | 0.216 | 0.824 | 0.182 | 0.261 | 0.464 | 0.576 | 0.634 | 0.351 | 0.600 | 0.500 | 1.000 | 0.681 | 0.195 | 0.819 |
| Jiangsu | 1.000 | 1.000 | 1.000 | 1.000 | 0.997 | 1.000 | 0.588 | 0.192 | 0.767 | 0.864 | 0.753 | 0.435 | 0.444 | 0.800 | 0.583 | 0.615 | 1.000 | 0.703 | 0.163 |
| Zhejiang | 0.226 | 0.633 | 0.566 | 0.305 | 0.795 | 0.721 | 0.176 | 0.046 | 0.668 | 0.534 | 0.686 | 0.427 | 0.330 | 0.200 | 0.500 | 0.222 | 0.411 | 0.551 | 0.158 |
| Anhui | 0.355 | 0.233 | 0.602 | 0.172 | 0.737 | 0.523 | 0.000 | 0.028 | 0.222 | 0.253 | 0.373 | 0.330 | 0.164 | 0.200 | 0.250 | 0.171 | 0.299 | 0.167 | 0.369 |
| Fujian | 0.419 | 0.562 | 0.703 | 0.309 | 1.000 | 0.630 | 0.000 | 0.003 | 0.211 | 0.241 | 0.405 | 0.272 | 0.271 | 0.000 | 0.333 | 0.162 | 0.198 | 0.204 | 0.000 |
| Jiangxi | 0.065 | 0.188 | 0.295 | 0.171 | 0.194 | 0.325 | 0.118 | 0.074 | 0.141 | 0.123 | 0.223 | 0.233 | 0.262 | 0.200 | 0.333 | 0.043 | 0.184 | 0.111 | 0.433 |
| Shandong | 0.323 | 0.317 | 0.853 | 0.281 | 0.695 | 0.728 | 0.235 | 0.350 | 0.390 | 0.483 | 0.534 | 0.333 | 0.583 | 0.200 | 1.000 | 0.291 | 0.545 | 0.335 | 0.435 |
| Henan | 0.516 | 0.332 | 0.789 | 0.403 | 0.412 | 0.666 | 0.176 | 0.145 | 0.231 | 0.258 | 0.320 | 0.227 | 0.253 | 0.200 | 0.333 | 0.094 | 0.353 | 0.171 | 0.294 |
| Hubei | 0.484 | 0.250 | 0.407 | 0.443 | 0.306 | 0.407 | 0.294 | 0.170 | 0.219 | 0.288 | 0.455 | 0.335 | 0.188 | 0.200 | 0.750 | 0.154 | 0.555 | 0.153 | 1.000 |
| Hunan | 0.548 | 0.326 | 0.525 | 0.249 | 0.296 | 0.287 | 0.059 | 0.064 | 0.202 | 0.257 | 0.471 | 0.309 | 0.258 | 0.000 | 0.333 | 0.051 | 0.428 | 0.109 | 0.226 |
| Guangdong | 0.516 | 0.396 | 0.697 | 0.396 | 0.748 | 0.752 | 0.353 | 0.098 | 1.000 | 1.000 | 0.819 | 0.469 | 1.000 | 1.000 | 0.750 | 0.487 | 0.823 | 1.000 | 0.447 |
| Guangxi | 0.097 | 0.142 | 0.254 | 0.059 | 0.231 | 0.154 | 0.176 | 0.005 | 0.051 | 0.049 | 0.247 | 0.089 | 0.313 | 0.200 | 0.250 | 0.068 | 0.183 | 0.046 | 0.250 |
| Hainan | 0.000 | 0.002 | 0.032 | 0.003 | 0.005 | 0.000 | 0.118 | 0.049 | 0.008 | 0.009 | 0.034 | 0.069 | 0.008 | 0.000 | 0.167 | 0.026 | 0.031 | 0.010 | 0.456 |
| Chongqing | 0.323 | 0.249 | 0.435 | 0.226 | 0.096 | 0.298 | 0.176 | 0.000 | 0.120 | 0.150 | 0.265 | 0.303 | 0.413 | 0.200 | 0.417 | 0.060 | 0.248 | 0.076 | 0.174 |
| Sichuan | 0.387 | 0.457 | 0.637 | 0.367 | 0.653 | 0.665 | 0.118 | 0.022 | 0.216 | 0.302 | 0.610 | 0.312 | 0.332 | 0.800 | 0.417 | 0.179 | 0.534 | 0.151 | 0.120 |
| Guizhou | 0.065 | 0.089 | 0.207 | 0.093 | 0.124 | 0.141 | 0.059 | 0.045 | 0.046 | 0.045 | 0.159 | 0.110 | 0.200 | 0.000 | 0.083 | 0.068 | 0.139 | 0.047 | 0.321 |
| Yunnan | 0.129 | 0.154 | 0.408 | 0.168 | 0.161 | 0.297 | 0.118 | 0.082 | 0.068 | 0.070 | 0.165 | 0.124 | 0.343 | 0.000 | 0.250 | 0.068 | 0.147 | 0.038 | 0.156 |
| Tibet | 0.000 | 0.000 | 0.053 | 0.006 | 0.039 | 0.013 | 0.000 | 0.166 | 0.000 | 0.000 | 0.001 | 0.000 | 0.020 | 0.200 | 0.000 | 0.000 | 0.000 | 0.000 | 0.393 |
| Shaanxi | 0.484 | 0.162 | 0.370 | 0.156 | 0.641 | 0.294 | 0.059 | 0.130 | 0.135 | 0.181 | 0.536 | 0.353 | 0.119 | 0.000 | 0.417 | 0.068 | 0.518 | 0.083 | 0.468 |
| Gansu | 0.161 | 0.051 | 0.040 | 0.032 | 0.184 | 0.160 | 0.118 | 0.513 | 0.029 | 0.030 | 0.106 | 0.159 | 0.191 | 0.000 | 0.167 | 0.051 | 0.113 | 0.027 | 0.212 |
| Qinghai | 0.000 | 0.002 | 0.004 | 0.000 | 0.019 | 0.018 | 0.000 | 0.411 | 0.003 | 0.005 | 0.000 | 0.077 | 0.000 | 0.000 | 0.083 | 0.000 | 0.019 | 0.004 | 0.505 |
| Ningxia | 0.000 | 0.008 | 0.000 | 0.001 | 0.000 | 0.045 | 0.000 | 0.049 | 0.012 | 0.016 | 0.034 | 0.208 | 0.022 | 0.000 | 0.250 | 0.026 | 0.034 | 0.008 | 0.035 |
| Xinjiang | 0.032 | 0.039 | 0.142 | 0.029 | 0.057 | 0.114 | 0.059 | 0.066 | 0.014 | 0.016 | 0.073 | 0.035 | 0.144 | 0.200 | 0.083 | 0.017 | 0.072 | 0.016 | 0.280 |

S1-10 CII Standardization Data in 2021

| **Scope of research** | **C1** | **C2** | **C3** | **C4** | **C5** | **C6** | **C7** | **C8** | **C9** | **C10** | **C11** | **C12** | **C13** | **C14** | **C15** | **C16** | **C17** | **C18** | **C19** |
| --- | --- | --- | --- | --- | --- | --- | --- | --- | --- | --- | --- | --- | --- | --- | --- | --- | --- | --- | --- |
| Beijing | 0.583 | 0.060 | 0.186 | 0.665 | 0.292 | 0.202 | 0.900 | 0.808 | 0.381 | 0.656 | 1.000 | 1.000 | 0.376 | 0.500 | 0.800 | 0.402 | 0.863 | 0.226 | 0.724 |
| Tianjin | 0.250 | 0.061 | 0.061 | 0.055 | 0.043 | 0.190 | 0.000 | 1.000 | 0.115 | 0.142 | 0.235 | 0.540 | 0.110 | 0.250 | 0.100 | 0.393 | 0.247 | 0.110 | 0.466 |
| Hebei | 0.458 | 0.086 | 0.286 | 0.063 | 0.271 | 0.258 | 0.000 | 0.469 | 0.140 | 0.185 | 0.258 | 0.250 | 0.134 | 0.000 | 0.300 | 0.094 | 0.219 | 0.136 | 0.736 |
| Shanxi | 0.083 | 0.099 | 0.105 | 0.095 | 0.152 | 0.311 | 0.050 | 0.558 | 0.063 | 0.062 | 0.157 | 0.133 | 0.148 | 0.000 | 0.400 | 0.017 | 0.164 | 0.041 | 0.318 |
| Inner Mongolia | 0.125 | 0.013 | 0.163 | 0.006 | 0.036 | 0.068 | 0.100 | 0.808 | 0.028 | 0.046 | 0.098 | 0.103 | 0.167 | 0.250 | 0.200 | 0.051 | 0.077 | 0.026 | 0.454 |
| Liaoning | 0.313 | 0.060 | 0.190 | 0.014 | 0.198 | 0.498 | 0.050 | 0.324 | 0.130 | 0.149 | 0.303 | 0.303 | 0.053 | 0.000 | 0.100 | 0.128 | 0.371 | 0.090 | 0.584 |
| Jilin | 0.250 | 0.034 | 0.090 | 0.031 | 0.136 | 0.227 | 0.050 | 0.323 | 0.056 | 0.044 | 0.254 | 0.176 | 0.204 | 0.000 | 0.100 | 0.017 | 0.233 | 0.032 | 0.630 |
| Heilongjiang | 0.250 | 0.018 | 0.014 | 0.000 | 0.085 | 0.173 | 0.000 | 0.793 | 0.053 | 0.047 | 0.192 | 0.163 | 0.334 | 0.000 | 0.100 | 0.060 | 0.262 | 0.042 | 0.240 |
| Shanghai | 0.229 | 0.081 | 0.066 | 0.134 | 0.170 | 0.188 | 1.000 | 0.584 | 0.265 | 0.454 | 0.611 | 0.628 | 0.332 | 1.000 | 0.500 | 1.000 | 0.780 | 0.204 | 0.998 |
| Jiangsu | 1.000 | 1.000 | 1.000 | 1.000 | 0.846 | 1.000 | 0.400 | 0.302 | 0.854 | 0.859 | 0.803 | 0.426 | 0.405 | 1.000 | 0.800 | 0.615 | 1.000 | 0.734 | 0.108 |
| Zhejiang | 0.313 | 0.610 | 0.567 | 0.295 | 0.857 | 0.762 | 0.250 | 0.076 | 0.649 | 0.538 | 0.675 | 0.425 | 0.311 | 0.250 | 1.000 | 0.222 | 0.439 | 0.533 | 0.162 |
| Anhui | 0.313 | 0.220 | 0.616 | 0.176 | 0.821 | 0.590 | 0.050 | 0.108 | 0.265 | 0.250 | 0.434 | 0.329 | 0.141 | 0.250 | 0.500 | 0.171 | 0.322 | 0.174 | 0.480 |
| Fujian | 0.438 | 0.541 | 0.701 | 0.319 | 1.000 | 0.673 | 0.050 | 0.000 | 0.265 | 0.241 | 0.390 | 0.271 | 0.260 | 0.000 | 0.400 | 0.162 | 0.204 | 0.175 | 0.000 |
| Jiangxi | 0.167 | 0.183 | 0.303 | 0.174 | 0.228 | 0.395 | 0.000 | 0.156 | 0.139 | 0.124 | 0.253 | 0.226 | 0.248 | 0.000 | 0.500 | 0.043 | 0.185 | 0.110 | 0.490 |
| Shandong | 0.396 | 0.305 | 0.841 | 0.308 | 0.542 | 0.811 | 0.100 | 0.277 | 0.505 | 0.485 | 0.591 | 0.329 | 0.528 | 0.500 | 0.800 | 0.291 | 0.566 | 0.377 | 0.484 |
| Henan | 0.396 | 0.325 | 0.771 | 0.380 | 0.343 | 0.709 | 0.550 | 0.255 | 0.250 | 0.253 | 0.326 | 0.231 | 0.246 | 0.250 | 0.500 | 0.094 | 0.369 | 0.179 | 0.316 |
| Hubei | 0.500 | 0.261 | 0.436 | 0.448 | 0.304 | 0.432 | 0.150 | 0.385 | 0.259 | 0.289 | 0.515 | 0.325 | 0.175 | 0.250 | 0.600 | 0.154 | 0.568 | 0.176 | 1.000 |
| Hunan | 0.500 | 0.314 | 0.531 | 0.257 | 0.292 | 0.298 | 0.200 | 0.100 | 0.235 | 0.256 | 0.486 | 0.311 | 0.216 | 0.250 | 0.500 | 0.051 | 0.397 | 0.111 | 0.270 |
| Guangdong | 0.479 | 0.400 | 0.706 | 0.314 | 0.673 | 0.740 | 0.150 | 0.463 | 1.000 | 1.000 | 0.858 | 0.470 | 1.000 | 0.500 | 0.900 | 0.487 | 0.842 | 1.000 | 0.503 |
| Guangxi | 0.125 | 0.131 | 0.261 | 0.079 | 0.251 | 0.187 | 0.150 | 0.024 | 0.061 | 0.048 | 0.263 | 0.083 | 0.283 | 0.250 | 0.200 | 0.068 | 0.181 | 0.052 | 0.481 |
| Hainan | 0.000 | 0.003 | 0.032 | 0.006 | 0.005 | 0.000 | 0.050 | 0.041 | 0.013 | 0.010 | 0.053 | 0.071 | 0.009 | 0.000 | 0.200 | 0.026 | 0.035 | 0.013 | 0.802 |
| Chongqing | 0.292 | 0.230 | 0.432 | 0.211 | 0.111 | 0.290 | 0.250 | 0.013 | 0.138 | 0.150 | 0.279 | 0.300 | 0.401 | 0.500 | 0.300 | 0.060 | 0.256 | 0.085 | 0.260 |
| Sichuan | 0.313 | 0.412 | 0.631 | 0.383 | 0.541 | 0.685 | 0.100 | 0.057 | 0.221 | 0.302 | 0.600 | 0.316 | 0.301 | 0.500 | 0.600 | 0.179 | 0.558 | 0.167 | 0.223 |
| Guizhou | 0.042 | 0.077 | 0.191 | 0.035 | 0.013 | 0.155 | 0.000 | 0.086 | 0.047 | 0.044 | 0.169 | 0.101 | 0.193 | 0.250 | 0.000 | 0.068 | 0.135 | 0.043 | 0.499 |
| Yunnan | 0.104 | 0.133 | 0.394 | 0.120 | 0.144 | 0.315 | 0.100 | 0.296 | 0.065 | 0.069 | 0.168 | 0.120 | 0.313 | 0.250 | 0.400 | 0.068 | 0.140 | 0.045 | 0.296 |
| Tibet | 0.000 | 0.000 | 0.033 | 0.000 | 0.029 | 0.012 | 0.000 | 0.376 | 0.000 | 0.000 | 0.001 | 0.000 | 0.018 | 0.000 | 0.100 | 0.000 | 0.000 | 0.000 | 0.601 |
| Shaanxi | 0.417 | 0.144 | 0.341 | 0.178 | 0.522 | 0.302 | 0.100 | 0.261 | 0.140 | 0.174 | 0.561 | 0.330 | 0.108 | 0.500 | 0.300 | 0.068 | 0.496 | 0.097 | 0.527 |
| Gansu | 0.167 | 0.048 | 0.041 | 0.035 | 0.103 | 0.189 | 0.000 | 0.484 | 0.036 | 0.031 | 0.113 | 0.155 | 0.167 | 0.000 | 0.200 | 0.051 | 0.124 | 0.028 | 0.315 |
| Qinghai | 0.000 | 0.002 | 0.005 | 0.004 | 0.020 | 0.011 | 0.000 | 0.829 | 0.004 | 0.005 | 0.000 | 0.082 | 0.000 | 0.000 | 0.000 | 0.000 | 0.018 | 0.005 | 0.891 |
| Ningxia | 0.000 | 0.008 | 0.000 | 0.002 | 0.000 | 0.045 | 0.000 | 0.228 | 0.016 | 0.016 | 0.035 | 0.204 | 0.018 | 0.000 | 0.000 | 0.026 | 0.021 | 0.013 | 0.151 |
| Xinjiang | 0.042 | 0.036 | 0.150 | 0.034 | 0.027 | 0.130 | 0.000 | 0.258 | 0.020 | 0.018 | 0.073 | 0.032 | 0.124 | 0.000 | 0.400 | 0.017 | 0.092 | 0.022 | 0.398 |

## S2 RED Standardization Data

S2-1 RED Standardization Data in 2012

| **Scope of research** | V1 | V2 | V3 | V4 | V5 | V6 | V7 | V8 | V9 | V10 | V11 | V12 | V13 | V14 | V15 | V16 |
| --- | --- | --- | --- | --- | --- | --- | --- | --- | --- | --- | --- | --- | --- | --- | --- | --- |
| Beijing | 0.300 | 0.180 | 0.130 | 0.330 | 0.530 | 0.950 | 0.551 | 0.990 | 0.970 | 1.000 | 1.000 | 0.840 | 0.900 | 0.920 | 0.940 | 1.000 |
| Tianjin | 0.220 | 0.240 | 0.240 | 0.160 | 0.270 | 0.880 | 0.164 | 0.970 | 0.720 | 0.730 | 0.450 | 0.540 | 0.720 | 1.000 | 0.880 | 0.210 |
| Hebei | 0.460 | 0.620 | 0.480 | 0.400 | 0.330 | 0.360 | 0.333 | 0.530 | 0.390 | 0.150 | 0.120 | 0.150 | 0.270 | 0.230 | 0.000 | 0.180 |
| Shanxi | 0.200 | 0.270 | 0.230 | 0.190 | 0.230 | 0.430 | 0.142 | 0.790 | 0.620 | 0.250 | 0.150 | 0.140 | 0.140 | 0.190 | 0.080 | 0.300 |
| Inner Mongolia | 0.270 | 0.370 | 0.300 | 0.190 | 0.240 | 0.530 | 0.153 | 0.650 | 0.750 | 0.350 | 0.070 | 0.260 | 0.230 | 0.600 | 0.140 | 0.170 |
| Liaoning | 0.430 | 0.690 | 0.450 | 0.410 | 0.490 | 0.640 | 0.252 | 0.670 | 0.830 | 0.310 | 0.230 | 0.260 | 0.370 | 0.500 | 0.310 | 0.230 |
| Jilin | 0.200 | 0.290 | 0.210 | 0.200 | 0.160 | 0.470 | 0.142 | 0.540 | 0.850 | 0.330 | 0.120 | 0.130 | 0.310 | 0.320 | 0.740 | 0.200 |
| Heilongjiang | 0.230 | 0.300 | 0.200 | 0.230 | 0.180 | 0.510 | 0.128 | 0.390 | 0.800 | 0.360 | 0.140 | 0.030 | 0.310 | 0.220 | 0.530 | 0.030 |
| Shanghai | 0.350 | 0.150 | 0.270 | 0.320 | 0.600 | 1.000 | 0.474 | 1.000 | 1.000 | 0.740 | 0.550 | 1.000 | 1.000 | 0.890 | 0.880 | 0.400 |
| Jiangsu | 0.950 | 0.990 | 0.930 | 0.810 | 0.940 | 0.600 | 0.863 | 0.770 | 0.560 | 0.670 | 0.370 | 0.540 | 0.580 | 0.660 | 0.440 | 0.370 |
| Zhejiang | 0.600 | 0.560 | 0.590 | 0.590 | 0.550 | 0.610 | 0.577 | 0.830 | 0.790 | 0.480 | 0.320 | 0.760 | 0.760 | 0.590 | 0.660 | 0.410 |
| Anhui | 0.290 | 0.480 | 0.310 | 0.240 | 0.280 | 0.360 | 0.251 | 0.500 | 0.290 | 0.250 | 0.240 | 0.170 | 0.200 | 0.120 | 0.620 | 0.190 |
| Fujian | 0.340 | 0.380 | 0.330 | 0.310 | 0.280 | 0.550 | 0.287 | 0.650 | 0.510 | 0.350 | 0.200 | 0.470 | 0.410 | 0.450 | 0.760 | 0.200 |
| Jiangxi | 0.220 | 0.330 | 0.220 | 0.170 | 0.210 | 0.370 | 0.153 | 0.540 | 0.200 | 0.110 | 0.110 | 0.120 | 0.250 | 0.120 | 0.640 | 0.420 |
| Shandong | 0.870 | 1.000 | 0.880 | 0.870 | 0.650 | 0.450 | 0.738 | 0.670 | 0.420 | 0.350 | 0.310 | 0.370 | 0.370 | 0.440 | 0.050 | 0.300 |
| Henan | 0.510 | 0.680 | 0.580 | 0.480 | 0.320 | 0.300 | 0.374 | 0.500 | 0.220 | 0.200 | 0.140 | 0.140 | 0.230 | 0.160 | 0.270 | 0.390 |
| Hubei | 0.380 | 0.490 | 0.380 | 0.420 | 0.280 | 0.460 | 0.315 | 0.500 | 0.550 | 0.380 | 0.260 | 0.160 | 0.250 | 0.260 | 0.630 | 0.140 |
| Hunan | 0.380 | 0.450 | 0.350 | 0.340 | 0.280 | 0.360 | 0.313 | 0.470 | 0.190 | 0.280 | 0.180 | 0.180 | 0.220 | 0.190 | 0.620 | 0.000 |
| Guangdong | 1.000 | 0.590 | 1.000 | 1.000 | 1.000 | 0.670 | 1.000 | 0.820 | 0.640 | 0.670 | 0.340 | 0.570 | 0.450 | 0.470 | 0.560 | 0.590 |
| Guangxi | 0.220 | 0.300 | 0.200 | 0.190 | 0.180 | 0.310 | 0.161 | 0.340 | 0.020 | 0.150 | 0.090 | 0.180 | 0.110 | 0.110 | 0.690 | 0.280 |
| Hainan | 0.040 | 0.050 | 0.020 | 0.030 | 0.050 | 0.430 | 0.036 | 0.000 | 0.440 | 0.180 | 0.040 | 0.160 | 0.220 | 0.170 | 0.980 | 0.750 |
| Chongqing | 0.190 | 0.260 | 0.190 | 0.170 | 0.260 | 0.510 | 0.188 | 0.690 | 0.220 | 0.290 | 0.200 | 0.250 | 0.220 | 0.260 | 0.710 | 0.310 |
| Sichuan | 0.410 | 0.540 | 0.410 | 0.400 | 0.380 | 0.310 | 0.344 | 0.460 | 0.320 | 0.150 | 0.210 | 0.140 | 0.190 | 0.130 | 0.550 | 0.070 |
| Guizhou | 0.110 | 0.170 | 0.080 | 0.080 | 0.150 | 0.210 | 0.104 | 0.490 | 0.000 | 0.190 | 0.060 | 0.070 | 0.020 | 0.000 | 0.480 | 0.320 |
| Yunnan | 0.170 | 0.230 | 0.130 | 0.150 | 0.200 | 0.250 | 0.173 | 0.370 | 0.370 | 0.120 | 0.070 | 0.170 | 0.070 | 0.030 | 0.590 | 0.070 |
| Tibet | 0.000 | 0.000 | 0.000 | 0.000 | 0.000 | 0.000 | 0.000 | 0.550 | 0.360 | 0.000 | 0.000 | 0.040 | 0.090 | 0.040 | 1.000 | 0.560 |
| Shaanxi | 0.240 | 0.370 | 0.260 | 0.180 | 0.250 | 0.410 | 0.181 | 0.640 | 0.650 | 0.480 | 0.300 | 0.160 | 0.090 | 0.260 | 0.500 | 0.340 |
| Gansu | 0.090 | 0.150 | 0.080 | 0.070 | 0.070 | 0.240 | 0.072 | 0.460 | 0.480 | 0.190 | 0.140 | 0.000 | 0.000 | 0.030 | 0.700 | 0.520 |
| Qinghai | 0.020 | 0.040 | 0.030 | 0.010 | 0.020 | 0.370 | 0.012 | 0.640 | 0.350 | 0.100 | 0.080 | 0.020 | 0.060 | 0.180 | 0.880 | 0.290 |
| Ningxia | 0.030 | 0.050 | 0.030 | 0.010 | 0.030 | 0.420 | 0.020 | 0.680 | 0.320 | 0.280 | 0.090 | 0.120 | 0.130 | 0.230 | 0.770 | 0.020 |
| Xinjiang | 0.120 | 0.180 | 0.110 | 0.070 | 0.130 | 0.320 | 0.093 | 0.300 | 0.370 | 0.150 | 0.050 | 0.030 | 0.140 | 0.190 | 0.420 | 0.280 |

S2-2 RED Standardization Data in 2013

| **Scope of research** | V1 | V2 | V3 | V4 | V5 | V6 | V7 | V8 | V9 | V10 | V11 | V12 | V13 | V14 | V15 | V16 |
| --- | --- | --- | --- | --- | --- | --- | --- | --- | --- | --- | --- | --- | --- | --- | --- | --- |
| Beijing | 0.300 | 0.170 | 0.130 | 0.320 | 0.510 | 0.950 | 0.548 | 0.990 | 1.000 | 1.000 | 1.000 | 0.990 | 0.850 | 0.930 | 0.950 | 1.000 |
| Tianjin | 0.220 | 0.230 | 0.250 | 0.170 | 0.280 | 0.880 | 0.165 | 0.970 | 0.740 | 0.700 | 0.470 | 0.360 | 0.720 | 1.000 | 0.890 | 0.260 |
| Hebei | 0.450 | 0.620 | 0.490 | 0.410 | 0.310 | 0.370 | 0.317 | 0.500 | 0.310 | 0.160 | 0.120 | 0.090 | 0.260 | 0.200 | 0.000 | 0.240 |
| Shanxi | 0.190 | 0.280 | 0.220 | 0.190 | 0.230 | 0.440 | 0.139 | 0.760 | 0.670 | 0.230 | 0.160 | 0.100 | 0.170 | 0.150 | 0.120 | 0.410 |
| Inner Mongolia | 0.260 | 0.370 | 0.290 | 0.190 | 0.230 | 0.530 | 0.150 | 0.620 | 0.710 | 0.360 | 0.070 | 0.250 | 0.250 | 0.580 | 0.160 | 0.240 |
| Liaoning | 0.430 | 0.670 | 0.460 | 0.410 | 0.470 | 0.650 | 0.253 | 0.660 | 0.740 | 0.290 | 0.240 | 0.270 | 0.340 | 0.500 | 0.350 | 0.340 |
| Jilin | 0.200 | 0.250 | 0.220 | 0.200 | 0.150 | 0.460 | 0.139 | 0.530 | 0.740 | 0.260 | 0.110 | 0.060 | 0.310 | 0.320 | 0.730 | 0.230 |
| Heilongjiang | 0.220 | 0.290 | 0.190 | 0.240 | 0.170 | 0.510 | 0.122 | 0.280 | 0.850 | 0.330 | 0.150 | 0.040 | 0.280 | 0.190 | 0.540 | 0.000 |
| Shanghai | 0.340 | 0.130 | 0.260 | 0.310 | 0.570 | 1.000 | 0.480 | 1.000 | 0.930 | 0.750 | 0.570 | 1.000 | 1.000 | 0.880 | 0.890 | 0.140 |
| Jiangsu | 0.950 | 0.990 | 0.950 | 0.810 | 0.930 | 0.610 | 0.882 | 0.760 | 0.480 | 0.700 | 0.380 | 0.470 | 0.580 | 0.680 | 0.450 | 0.440 |
| Zhejiang | 0.600 | 0.550 | 0.590 | 0.590 | 0.530 | 0.610 | 0.570 | 0.820 | 0.800 | 0.510 | 0.330 | 0.690 | 0.870 | 0.590 | 0.650 | 0.440 |
| Anhui | 0.300 | 0.490 | 0.330 | 0.250 | 0.280 | 0.370 | 0.258 | 0.500 | 0.280 | 0.260 | 0.270 | 0.120 | 0.240 | 0.120 | 0.650 | 0.320 |
| Fujian | 0.340 | 0.400 | 0.350 | 0.320 | 0.290 | 0.560 | 0.284 | 0.650 | 0.530 | 0.360 | 0.200 | 0.330 | 0.430 | 0.450 | 0.760 | 0.280 |
| Jiangxi | 0.220 | 0.330 | 0.240 | 0.170 | 0.220 | 0.380 | 0.153 | 0.540 | 0.130 | 0.120 | 0.110 | 0.090 | 0.260 | 0.110 | 0.650 | 0.390 |
| Shandong | 0.880 | 1.000 | 0.900 | 0.870 | 0.640 | 0.460 | 0.740 | 0.660 | 0.370 | 0.360 | 0.320 | 0.280 | 0.370 | 0.440 | 0.100 | 0.370 |
| Henan | 0.510 | 0.700 | 0.550 | 0.480 | 0.330 | 0.300 | 0.380 | 0.490 | 0.160 | 0.220 | 0.140 | 0.070 | 0.250 | 0.140 | 0.270 | 0.420 |
| Hubei | 0.390 | 0.510 | 0.380 | 0.420 | 0.300 | 0.470 | 0.341 | 0.490 | 0.450 | 0.380 | 0.260 | 0.110 | 0.300 | 0.260 | 0.630 | 0.290 |
| Hunan | 0.390 | 0.470 | 0.370 | 0.350 | 0.280 | 0.370 | 0.321 | 0.490 | 0.200 | 0.270 | 0.180 | 0.180 | 0.250 | 0.180 | 0.620 | 0.070 |
| Guangdong | 1.000 | 0.600 | 1.000 | 1.000 | 1.000 | 0.670 | 1.000 | 0.820 | 0.570 | 0.610 | 0.350 | 0.390 | 0.400 | 0.460 | 0.570 | 0.620 |
| Guangxi | 0.220 | 0.310 | 0.210 | 0.190 | 0.170 | 0.320 | 0.167 | 0.330 | 0.000 | 0.130 | 0.080 | 0.110 | 0.160 | 0.100 | 0.710 | 0.350 |
| Hainan | 0.040 | 0.050 | 0.020 | 0.030 | 0.060 | 0.440 | 0.039 | 0.000 | 0.410 | 0.150 | 0.030 | 0.100 | 0.240 | 0.160 | 0.980 | 0.700 |
| Chongqing | 0.190 | 0.270 | 0.170 | 0.170 | 0.230 | 0.530 | 0.189 | 0.680 | 0.190 | 0.290 | 0.190 | 0.130 | 0.210 | 0.260 | 0.720 | 0.320 |
| Sichuan | 0.410 | 0.540 | 0.430 | 0.410 | 0.380 | 0.320 | 0.348 | 0.470 | 0.110 | 0.160 | 0.210 | 0.090 | 0.200 | 0.120 | 0.570 | 0.100 |
| Guizhou | 0.120 | 0.180 | 0.100 | 0.080 | 0.160 | 0.210 | 0.112 | 0.480 | 0.010 | 0.210 | 0.050 | 0.030 | 0.020 | 0.000 | 0.510 | 0.370 |
| Yunnan | 0.180 | 0.250 | 0.140 | 0.150 | 0.220 | 0.250 | 0.184 | 0.340 | 0.330 | 0.100 | 0.070 | 0.100 | 0.080 | 0.030 | 0.600 | 0.140 |
| Tibet | 0.000 | 0.000 | 0.000 | 0.000 | 0.000 | 0.000 | 0.000 | 0.570 | 0.120 | 0.000 | 0.000 | 0.020 | 0.070 | 0.040 | 1.000 | 0.610 |
| Shaanxi | 0.250 | 0.390 | 0.280 | 0.190 | 0.240 | 0.420 | 0.186 | 0.620 | 0.510 | 0.510 | 0.320 | 0.100 | 0.110 | 0.260 | 0.480 | 0.350 |
| Gansu | 0.090 | 0.160 | 0.080 | 0.070 | 0.070 | 0.250 | 0.074 | 0.430 | 0.520 | 0.180 | 0.140 | 0.000 | 0.000 | 0.020 | 0.700 | 0.660 |
| Qinghai | 0.020 | 0.040 | 0.030 | 0.010 | 0.020 | 0.380 | 0.012 | 0.600 | 0.520 | 0.090 | 0.060 | 0.020 | 0.060 | 0.180 | 0.880 | 0.350 |
| Ningxia | 0.030 | 0.050 | 0.030 | 0.010 | 0.030 | 0.430 | 0.020 | 0.650 | 0.430 | 0.280 | 0.090 | 0.060 | 0.150 | 0.210 | 0.760 | 0.120 |
| Xinjiang | 0.120 | 0.190 | 0.110 | 0.070 | 0.150 | 0.320 | 0.104 | 0.280 | 0.300 | 0.130 | 0.040 | 0.050 | 0.170 | 0.190 | 0.390 | 0.330 |

S2-3 RED Standardization Data in 2014

| **Scope of research** | V1 | V2 | V3 | V4 | V5 | V6 | V7 | V8 | V9 | V10 | V11 | V12 | V13 | V14 | V15 | V16 |
| --- | --- | --- | --- | --- | --- | --- | --- | --- | --- | --- | --- | --- | --- | --- | --- | --- |
| Beijing | 0.310 | 0.140 | 0.130 | 0.330 | 0.490 | 0.950 | 0.545 | 0.990 | 1.000 | 1.000 | 1.000 | 0.990 | 0.840 | 0.930 | 0.960 | 1.000 |
| Tianjin | 0.220 | 0.230 | 0.240 | 0.160 | 0.290 | 0.890 | 0.163 | 0.970 | 0.740 | 0.840 | 0.470 | 0.360 | 0.720 | 1.000 | 0.890 | 0.310 |
| Hebei | 0.430 | 0.620 | 0.460 | 0.410 | 0.290 | 0.370 | 0.307 | 0.500 | 0.310 | 0.530 | 0.140 | 0.090 | 0.260 | 0.170 | 0.000 | 0.280 |
| Shanxi | 0.180 | 0.270 | 0.190 | 0.190 | 0.210 | 0.440 | 0.136 | 0.750 | 0.670 | 0.080 | 0.160 | 0.080 | 0.170 | 0.110 | 0.090 | 0.340 |
| Inner Mongolia | 0.250 | 0.400 | 0.270 | 0.190 | 0.220 | 0.530 | 0.149 | 0.620 | 0.710 | 0.330 | 0.070 | 0.240 | 0.250 | 0.570 | 0.220 | 0.280 |
| Liaoning | 0.410 | 0.570 | 0.430 | 0.410 | 0.390 | 0.650 | 0.259 | 0.670 | 0.740 | 0.310 | 0.220 | 0.270 | 0.330 | 0.490 | 0.290 | 0.340 |
| Jilin | 0.190 | 0.250 | 0.220 | 0.200 | 0.140 | 0.460 | 0.133 | 0.530 | 0.740 | 0.250 | 0.120 | 0.050 | 0.300 | 0.300 | 0.720 | 0.340 |
| Heilongjiang | 0.210 | 0.210 | 0.160 | 0.240 | 0.150 | 0.510 | 0.124 | 0.250 | 0.850 | 0.330 | 0.140 | 0.030 | 0.280 | 0.160 | 0.580 | 0.000 |
| Shanghai | 0.340 | 0.120 | 0.250 | 0.320 | 0.560 | 1.000 | 0.489 | 1.000 | 0.930 | 0.710 | 0.600 | 1.000 | 1.000 | 0.900 | 0.900 | 0.130 |
| Jiangsu | 0.960 | 0.990 | 0.920 | 0.820 | 0.900 | 0.620 | 0.907 | 0.780 | 0.480 | 0.750 | 0.400 | 0.460 | 0.580 | 0.700 | 0.440 | 0.460 |
| Zhejiang | 0.590 | 0.560 | 0.570 | 0.620 | 0.500 | 0.610 | 0.556 | 0.830 | 0.800 | 0.560 | 0.350 | 0.690 | 0.880 | 0.590 | 0.810 | 0.480 |
| Anhui | 0.300 | 0.500 | 0.320 | 0.270 | 0.260 | 0.370 | 0.266 | 0.520 | 0.280 | 0.270 | 0.290 | 0.110 | 0.240 | 0.100 | 0.620 | 0.400 |
| Fujian | 0.350 | 0.410 | 0.360 | 0.320 | 0.280 | 0.560 | 0.287 | 0.650 | 0.530 | 0.390 | 0.210 | 0.330 | 0.430 | 0.470 | 0.760 | 0.320 |
| Jiangxi | 0.220 | 0.340 | 0.230 | 0.180 | 0.220 | 0.380 | 0.161 | 0.550 | 0.130 | 0.110 | 0.130 | 0.090 | 0.260 | 0.100 | 0.670 | 0.380 |
| Shandong | 0.870 | 1.000 | 0.870 | 0.880 | 0.620 | 0.460 | 0.775 | 0.670 | 0.370 | 0.360 | 0.340 | 0.270 | 0.380 | 0.440 | 0.060 | 0.370 |
| Henan | 0.510 | 0.720 | 0.540 | 0.490 | 0.330 | 0.300 | 0.395 | 0.500 | 0.160 | 0.230 | 0.160 | 0.070 | 0.250 | 0.140 | 0.310 | 0.470 |
| Hubei | 0.400 | 0.530 | 0.380 | 0.430 | 0.310 | 0.470 | 0.358 | 0.510 | 0.450 | 0.390 | 0.280 | 0.110 | 0.310 | 0.260 | 0.640 | 0.430 |
| Hunan | 0.390 | 0.490 | 0.370 | 0.370 | 0.270 | 0.370 | 0.332 | 0.510 | 0.200 | 0.280 | 0.190 | 0.180 | 0.250 | 0.180 | 0.630 | 0.100 |
| Guangdong | 1.000 | 0.610 | 1.000 | 1.000 | 1.000 | 0.660 | 1.000 | 0.820 | 0.570 | 0.600 | 0.370 | 0.380 | 0.400 | 0.470 | 0.610 | 0.640 |
| Guangxi | 0.220 | 0.310 | 0.210 | 0.190 | 0.160 | 0.320 | 0.168 | 0.340 | 0.000 | 0.140 | 0.080 | 0.110 | 0.160 | 0.080 | 0.710 | 0.420 |
| Hainan | 0.040 | 0.050 | 0.020 | 0.030 | 0.050 | 0.440 | 0.039 | 0.000 | 0.410 | 0.170 | 0.040 | 0.100 | 0.240 | 0.160 | 0.990 | 0.700 |
| Chongqing | 0.200 | 0.270 | 0.180 | 0.190 | 0.230 | 0.530 | 0.193 | 0.690 | 0.190 | 0.330 | 0.200 | 0.120 | 0.220 | 0.270 | 0.750 | 0.320 |
| Sichuan | 0.410 | 0.540 | 0.410 | 0.430 | 0.370 | 0.320 | 0.360 | 0.480 | 0.110 | 0.160 | 0.230 | 0.090 | 0.210 | 0.110 | 0.590 | 0.100 |
| Guizhou | 0.120 | 0.190 | 0.110 | 0.090 | 0.160 | 0.220 | 0.115 | 0.410 | 0.010 | 0.180 | 0.060 | 0.030 | 0.030 | 0.000 | 0.570 | 0.380 |
| Yunnan | 0.180 | 0.250 | 0.130 | 0.150 | 0.200 | 0.250 | 0.187 | 0.340 | 0.330 | 0.110 | 0.070 | 0.090 | 0.080 | 0.010 | 0.670 | 0.160 |
| Tibet | 0.000 | 0.000 | 0.000 | 0.000 | 0.000 | 0.000 | 0.000 | 0.580 | 0.120 | 0.000 | 0.000 | 0.010 | 0.070 | 0.040 | 1.000 | 0.630 |
| Shaanxi | 0.250 | 0.390 | 0.270 | 0.200 | 0.220 | 0.420 | 0.191 | 0.630 | 0.510 | 0.540 | 0.320 | 0.090 | 0.110 | 0.260 | 0.500 | 0.360 |
| Gansu | 0.090 | 0.160 | 0.080 | 0.080 | 0.070 | 0.250 | 0.075 | 0.440 | 0.520 | 0.200 | 0.150 | 0.000 | 0.000 | 0.000 | 0.700 | 0.720 |
| Qinghai | 0.020 | 0.040 | 0.030 | 0.010 | 0.020 | 0.380 | 0.012 | 0.610 | 0.520 | 0.090 | 0.060 | 0.020 | 0.070 | 0.170 | 0.870 | 0.420 |
| Ningxia | 0.030 | 0.050 | 0.030 | 0.010 | 0.030 | 0.440 | 0.018 | 0.670 | 0.430 | 0.300 | 0.110 | 0.050 | 0.140 | 0.200 | 0.800 | 0.140 |
| Xinjiang | 0.120 | 0.200 | 0.110 | 0.070 | 0.150 | 0.320 | 0.107 | 0.290 | 0.300 | 0.120 | 0.050 | 0.050 | 0.160 | 0.180 | 0.440 | 0.410 |

S2-4 RED Standardization Data in 2015

| **Scope of research** | V1 | V2 | V3 | V4 | V5 | V6 | V7 | V8 | V9 | V10 | V11 | V12 | V13 | V14 | V15 | V16 |
| --- | --- | --- | --- | --- | --- | --- | --- | --- | --- | --- | --- | --- | --- | --- | --- | --- |
| Beijing | 0.310 | 0.130 | 0.120 | 0.330 | 0.500 | 0.980 | 0.494 | 0.990 | 0.970 | 0.960 | 1.000 | 1.000 | 0.840 | 0.980 | 0.960 | 1.000 |
| Tianjin | 0.220 | 0.220 | 0.230 | 0.160 | 0.270 | 0.920 | 0.153 | 0.960 | 1.000 | 1.000 | 0.490 | 0.350 | 0.710 | 1.000 | 0.900 | 0.310 |
| Hebei | 0.400 | 0.600 | 0.420 | 0.410 | 0.270 | 0.390 | 0.308 | 0.510 | 0.370 | 0.210 | 0.150 | 0.080 | 0.250 | 0.170 | 0.000 | 0.280 |
| Shanxi | 0.160 | 0.270 | 0.140 | 0.190 | 0.160 | 0.460 | 0.168 | 0.750 | 0.720 | 0.220 | 0.130 | 0.070 | 0.150 | 0.110 | 0.040 | 0.310 |
| Inner Mongolia | 0.230 | 0.260 | 0.250 | 0.190 | 0.200 | 0.540 | 0.180 | 0.620 | 0.840 | 0.440 | 0.080 | 0.230 | 0.240 | 0.550 | 0.220 | 0.270 |
| Liaoning | 0.390 | 0.350 | 0.370 | 0.410 | 0.220 | 0.660 | 0.335 | 0.650 | 0.780 | 0.290 | 0.170 | 0.250 | 0.310 | 0.480 | 0.270 | 0.340 |
| Jilin | 0.180 | 0.240 | 0.200 | 0.200 | 0.120 | 0.460 | 0.117 | 0.520 | 0.820 | 0.250 | 0.120 | 0.040 | 0.270 | 0.300 | 0.700 | 0.310 |
| Heilongjiang | 0.200 | 0.190 | 0.130 | 0.240 | 0.110 | 0.520 | 0.121 | 0.250 | 0.930 | 0.290 | 0.130 | 0.010 | 0.260 | 0.160 | 0.590 | 0.000 |
| Shanghai | 0.340 | 0.110 | 0.230 | 0.320 | 0.580 | 1.000 | 0.480 | 1.000 | 0.870 | 0.780 | 0.600 | 1.000 | 1.000 | 0.950 | 0.900 | 0.140 |
| Jiangsu | 0.960 | 0.960 | 0.930 | 0.820 | 0.860 | 0.650 | 0.900 | 0.770 | 0.550 | 0.820 | 0.400 | 0.460 | 0.570 | 0.760 | 0.450 | 0.480 |
| Zhejiang | 0.580 | 0.550 | 0.570 | 0.620 | 0.510 | 0.640 | 0.561 | 0.830 | 0.720 | 0.660 | 0.360 | 0.680 | 0.870 | 0.630 | 0.680 | 0.500 |
| Anhui | 0.290 | 0.490 | 0.300 | 0.270 | 0.250 | 0.380 | 0.271 | 0.530 | 0.320 | 0.290 | 0.290 | 0.110 | 0.240 | 0.120 | 0.620 | 0.430 |
| Fujian | 0.350 | 0.430 | 0.360 | 0.320 | 0.260 | 0.580 | 0.272 | 0.660 | 0.530 | 0.360 | 0.210 | 0.330 | 0.420 | 0.510 | 0.750 | 0.260 |
| Jiangxi | 0.220 | 0.340 | 0.230 | 0.180 | 0.220 | 0.400 | 0.166 | 0.550 | 0.180 | 0.140 | 0.130 | 0.090 | 0.260 | 0.130 | 0.630 | 0.360 |
| Shandong | 0.860 | 1.000 | 0.860 | 0.880 | 0.580 | 0.490 | 0.755 | 0.670 | 0.400 | 0.390 | 0.340 | 0.270 | 0.370 | 0.460 | 0.030 | 0.360 |
| Henan | 0.500 | 0.730 | 0.520 | 0.490 | 0.310 | 0.320 | 0.379 | 0.520 | 0.150 | 0.240 | 0.150 | 0.060 | 0.240 | 0.160 | 0.260 | 0.490 |
| Hubei | 0.400 | 0.540 | 0.380 | 0.430 | 0.310 | 0.490 | 0.329 | 0.520 | 0.540 | 0.390 | 0.280 | 0.110 | 0.300 | 0.300 | 0.630 | 0.590 |
| Hunan | 0.390 | 0.510 | 0.360 | 0.370 | 0.260 | 0.390 | 0.339 | 0.510 | 0.260 | 0.340 | 0.200 | 0.170 | 0.250 | 0.200 | 0.610 | 0.120 |
| Guangdong | 1.000 | 0.620 | 1.000 | 1.000 | 1.000 | 0.680 | 1.000 | 0.820 | 0.780 | 0.610 | 0.380 | 0.380 | 0.390 | 0.510 | 0.620 | 0.660 |
| Guangxi | 0.220 | 0.320 | 0.210 | 0.190 | 0.150 | 0.320 | 0.170 | 0.350 | 0.000 | 0.140 | 0.060 | 0.090 | 0.160 | 0.110 | 0.720 | 0.500 |
| Hainan | 0.040 | 0.050 | 0.010 | 0.030 | 0.050 | 0.460 | 0.039 | 0.000 | 0.380 | 0.180 | 0.030 | 0.090 | 0.240 | 0.180 | 0.990 | 0.710 |
| Chongqing | 0.200 | 0.280 | 0.180 | 0.190 | 0.220 | 0.550 | 0.187 | 0.700 | 0.330 | 0.360 | 0.220 | 0.120 | 0.220 | 0.320 | 0.740 | 0.290 |
| Sichuan | 0.400 | 0.520 | 0.360 | 0.430 | 0.350 | 0.330 | 0.349 | 0.480 | 0.330 | 0.160 | 0.240 | 0.080 | 0.200 | 0.130 | 0.590 | 0.110 |
| Guizhou | 0.130 | 0.210 | 0.110 | 0.090 | 0.150 | 0.240 | 0.116 | 0.330 | 0.050 | 0.180 | 0.050 | 0.030 | 0.030 | 0.050 | 0.580 | 0.380 |
| Yunnan | 0.180 | 0.260 | 0.130 | 0.150 | 0.180 | 0.260 | 0.151 | 0.350 | 0.450 | 0.160 | 0.090 | 0.090 | 0.080 | 0.030 | 0.670 | 0.170 |
| Tibet | 0.000 | 0.000 | 0.000 | 0.000 | 0.000 | 0.000 | 0.000 | 0.600 | 0.290 | 0.000 | 0.000 | 0.060 | 0.080 | 0.070 | 1.000 | 0.650 |
| Shaanxi | 0.240 | 0.370 | 0.240 | 0.200 | 0.210 | 0.440 | 0.191 | 0.630 | 0.620 | 0.510 | 0.330 | 0.090 | 0.110 | 0.260 | 0.510 | 0.360 |
| Gansu | 0.080 | 0.160 | 0.060 | 0.080 | 0.070 | 0.260 | 0.076 | 0.400 | 0.540 | 0.090 | 0.160 | 0.000 | 0.000 | 0.000 | 0.680 | 0.760 |
| Qinghai | 0.020 | 0.040 | 0.030 | 0.010 | 0.010 | 0.380 | 0.013 | 0.640 | 0.480 | 0.060 | 0.030 | 0.030 | 0.060 | 0.180 | 0.860 | 0.420 |
| Ningxia | 0.030 | 0.050 | 0.030 | 0.010 | 0.030 | 0.460 | 0.020 | 0.660 | 0.450 | 0.310 | 0.100 | 0.050 | 0.130 | 0.220 | 0.790 | 0.150 |
| Xinjiang | 0.120 | 0.200 | 0.090 | 0.070 | 0.130 | 0.330 | 0.099 | 0.280 | 0.320 | 0.150 | 0.040 | 0.090 | 0.150 | 0.170 | 0.490 | 0.520 |

S2-5 RED Standardization Data in 2016

| **Scope of research** | V1 | V2 | V3 | V4 | V5 | V6 | V7 | V8 | V9 | V10 | V11 | V12 | V13 | V14 | V15 | V16 |
| --- | --- | --- | --- | --- | --- | --- | --- | --- | --- | --- | --- | --- | --- | --- | --- | --- |
| Beijing | 0.310 | 0.120 | 0.120 | 0.310 | 0.480 | 0.980 | 0.470 | 0.990 | 0.940 | 1.000 | 1.000 | 0.990 | 0.820 | 1.000 | 0.980 | 1.000 |
| Tianjin | 0.210 | 0.220 | 0.210 | 0.150 | 0.250 | 0.910 | 0.149 | 0.960 | 0.980 | 0.960 | 0.500 | 0.360 | 0.700 | 0.970 | 0.940 | 0.260 |
| Hebei | 0.390 | 0.580 | 0.410 | 0.410 | 0.260 | 0.410 | 0.298 | 0.540 | 0.280 | 0.190 | 0.180 | 0.080 | 0.250 | 0.170 | 0.000 | 0.190 |
| Shanxi | 0.150 | 0.240 | 0.120 | 0.180 | 0.140 | 0.460 | 0.155 | 0.760 | 0.810 | 0.210 | 0.150 | 0.050 | 0.150 | 0.090 | 0.340 | 0.250 |
| Inner Mongolia | 0.210 | 0.260 | 0.220 | 0.180 | 0.180 | 0.540 | 0.172 | 0.620 | 0.980 | 0.470 | 0.110 | 0.230 | 0.230 | 0.490 | 0.410 | 0.200 |
| Liaoning | 0.260 | 0.100 | 0.210 | 0.380 | 0.200 | 0.650 | 0.253 | 0.590 | 0.830 | 0.300 | 0.270 | 0.220 | 0.300 | 0.260 | 0.440 | 0.150 |
| Jilin | 0.170 | 0.240 | 0.180 | 0.200 | 0.110 | 0.450 | 0.112 | 0.580 | 0.860 | 0.250 | 0.130 | 0.030 | 0.260 | 0.290 | 0.810 | 0.270 |
| Heilongjiang | 0.180 | 0.180 | 0.110 | 0.230 | 0.100 | 0.510 | 0.114 | 0.260 | 1.000 | 0.310 | 0.140 | 0.000 | 0.240 | 0.140 | 0.620 | 0.000 |
| Shanghai | 0.340 | 0.100 | 0.230 | 0.310 | 0.610 | 1.000 | 0.484 | 1.000 | 0.940 | 0.830 | 0.630 | 1.000 | 1.000 | 0.980 | 0.930 | 0.050 |
| Jiangsu | 0.960 | 0.930 | 0.930 | 0.820 | 0.780 | 0.650 | 0.885 | 0.790 | 0.500 | 0.830 | 0.430 | 0.450 | 0.560 | 0.760 | 0.500 | 0.430 |
| Zhejiang | 0.580 | 0.550 | 0.570 | 0.630 | 0.500 | 0.640 | 0.550 | 0.840 | 0.760 | 0.670 | 0.390 | 0.670 | 0.850 | 0.630 | 0.790 | 0.480 |
| Anhui | 0.290 | 0.490 | 0.310 | 0.280 | 0.250 | 0.380 | 0.275 | 0.560 | 0.320 | 0.280 | 0.310 | 0.110 | 0.240 | 0.130 | 0.710 | 0.360 |
| Fujian | 0.350 | 0.420 | 0.360 | 0.330 | 0.240 | 0.580 | 0.273 | 0.660 | 0.400 | 0.360 | 0.250 | 0.320 | 0.420 | 0.520 | 0.800 | 0.130 |
| Jiangxi | 0.220 | 0.350 | 0.220 | 0.180 | 0.190 | 0.400 | 0.170 | 0.570 | 0.120 | 0.130 | 0.170 | 0.090 | 0.260 | 0.140 | 0.710 | 0.310 |
| Shandong | 0.840 | 1.000 | 0.840 | 0.880 | 0.560 | 0.500 | 0.730 | 0.700 | 0.360 | 0.370 | 0.380 | 0.260 | 0.360 | 0.450 | 0.020 | 0.270 |
| Henan | 0.490 | 0.750 | 0.520 | 0.500 | 0.290 | 0.320 | 0.381 | 0.560 | 0.120 | 0.220 | 0.180 | 0.050 | 0.230 | 0.160 | 0.590 | 0.430 |
| Hubei | 0.400 | 0.550 | 0.380 | 0.440 | 0.290 | 0.490 | 0.325 | 0.530 | 0.520 | 0.420 | 0.290 | 0.120 | 0.290 | 0.310 | 0.730 | 0.640 |
| Hunan | 0.380 | 0.520 | 0.350 | 0.380 | 0.250 | 0.400 | 0.338 | 0.520 | 0.200 | 0.340 | 0.230 | 0.170 | 0.250 | 0.210 | 0.710 | 0.010 |
| Guangdong | 1.000 | 0.610 | 1.000 | 1.000 | 1.000 | 0.680 | 1.000 | 0.820 | 0.780 | 0.620 | 0.420 | 0.370 | 0.390 | 0.510 | 0.700 | 0.620 |
| Guangxi | 0.220 | 0.320 | 0.210 | 0.190 | 0.140 | 0.320 | 0.166 | 0.350 | 0.100 | 0.120 | 0.080 | 0.080 | 0.160 | 0.110 | 0.780 | 0.460 |
| Hainan | 0.040 | 0.040 | 0.010 | 0.030 | 0.050 | 0.470 | 0.039 | 0.000 | 0.430 | 0.120 | 0.060 | 0.090 | 0.240 | 0.180 | 0.990 | 0.660 |
| Chongqing | 0.210 | 0.280 | 0.190 | 0.200 | 0.200 | 0.570 | 0.185 | 0.700 | 0.280 | 0.360 | 0.270 | 0.120 | 0.230 | 0.340 | 0.820 | 0.200 |
| Sichuan | 0.400 | 0.530 | 0.340 | 0.440 | 0.320 | 0.340 | 0.357 | 0.500 | 0.250 | 0.150 | 0.270 | 0.080 | 0.210 | 0.140 | 0.630 | 0.020 |
| Guizhou | 0.130 | 0.220 | 0.110 | 0.090 | 0.140 | 0.250 | 0.114 | 0.340 | 0.000 | 0.150 | 0.080 | 0.030 | 0.040 | 0.060 | 0.590 | 0.350 |
| Yunnan | 0.170 | 0.280 | 0.120 | 0.150 | 0.160 | 0.270 | 0.147 | 0.370 | 0.440 | 0.140 | 0.130 | 0.090 | 0.090 | 0.040 | 0.630 | 0.220 |
| Tibet | 0.000 | 0.000 | 0.000 | 0.000 | 0.000 | 0.000 | 0.000 | 0.580 | 0.320 | 0.000 | 0.000 | 0.070 | 0.090 | 0.080 | 1.000 | 0.580 |
| Shaanxi | 0.230 | 0.370 | 0.230 | 0.200 | 0.160 | 0.440 | 0.184 | 0.640 | 0.670 | 0.490 | 0.350 | 0.090 | 0.110 | 0.260 | 0.710 | 0.330 |
| Gansu | 0.080 | 0.160 | 0.050 | 0.080 | 0.060 | 0.260 | 0.072 | 0.420 | 0.540 | 0.170 | 0.180 | 0.000 | 0.000 | 0.000 | 0.790 | 0.720 |
| Qinghai | 0.020 | 0.040 | 0.030 | 0.010 | 0.010 | 0.380 | 0.013 | 0.640 | 0.530 | 0.060 | 0.060 | 0.030 | 0.070 | 0.180 | 0.880 | 0.390 |
| Ningxia | 0.030 | 0.040 | 0.030 | 0.010 | 0.020 | 0.460 | 0.019 | 0.690 | 0.540 | 0.320 | 0.130 | 0.050 | 0.130 | 0.220 | 0.790 | 0.110 |
| Xinjiang | 0.110 | 0.170 | 0.080 | 0.070 | 0.110 | 0.320 | 0.089 | 0.270 | 0.240 | 0.130 | 0.070 | 0.090 | 0.150 | 0.140 | 0.550 | 0.620 |

S2-6 RED Standardization Data in 2017

| **Scope of research** | V1 | V2 | V3 | V4 | V5 | V6 | V7 | V8 | V9 | V10 | V11 | V12 | V13 | V14 | V15 | V16 |
| --- | --- | --- | --- | --- | --- | --- | --- | --- | --- | --- | --- | --- | --- | --- | --- | --- |
| Beijing | 0.300 | 0.120 | 0.120 | 0.290 | 0.470 | 0.980 | 0.448 | 1.000 | 0.880 | 1.000 | 1.000 | 0.990 | 0.820 | 1.000 | 0.980 | 1.000 |
| Tianjin | 0.200 | 0.170 | 0.200 | 0.140 | 0.190 | 0.920 | 0.144 | 0.970 | 0.960 | 0.720 | 0.420 | 0.370 | 0.690 | 0.900 | 0.920 | 0.260 |
| Hebei | 0.370 | 0.590 | 0.390 | 0.410 | 0.270 | 0.420 | 0.294 | 0.580 | 0.260 | 0.190 | 0.200 | 0.090 | 0.240 | 0.170 | 0.000 | 0.190 |
| Shanxi | 0.160 | 0.080 | 0.120 | 0.170 | 0.150 | 0.470 | 0.150 | 0.800 | 0.770 | 0.230 | 0.140 | 0.050 | 0.140 | 0.130 | 0.290 | 0.280 |
| Inner Mongolia | 0.170 | 0.230 | 0.210 | 0.180 | 0.140 | 0.550 | 0.151 | 0.530 | 0.790 | 0.360 | 0.110 | 0.230 | 0.230 | 0.350 | 0.230 | 0.210 |
| Liaoning | 0.250 | 0.090 | 0.200 | 0.350 | 0.200 | 0.640 | 0.239 | 0.630 | 0.810 | 0.300 | 0.300 | 0.210 | 0.290 | 0.250 | 0.330 | 0.150 |
| Jilin | 0.150 | 0.210 | 0.180 | 0.190 | 0.090 | 0.450 | 0.106 | 0.670 | 0.770 | 0.220 | 0.120 | 0.020 | 0.250 | 0.260 | 0.750 | 0.250 |
| Heilongjiang | 0.170 | 0.180 | 0.100 | 0.230 | 0.090 | 0.500 | 0.106 | 0.140 | 1.000 | 0.230 | 0.130 | 0.000 | 0.230 | 0.130 | 0.510 | 0.000 |
| Shanghai | 0.330 | 0.100 | 0.230 | 0.300 | 0.580 | 1.000 | 0.463 | 1.000 | 0.810 | 0.750 | 0.690 | 1.000 | 1.000 | 0.980 | 0.960 | 0.100 |
| Jiangsu | 0.960 | 0.960 | 0.940 | 0.830 | 0.720 | 0.670 | 0.861 | 0.790 | 0.510 | 0.810 | 0.440 | 0.460 | 0.560 | 0.780 | 0.430 | 0.440 |
| Zhejiang | 0.570 | 0.560 | 0.580 | 0.630 | 0.500 | 0.650 | 0.545 | 0.840 | 0.770 | 0.690 | 0.410 | 0.680 | 0.850 | 0.630 | 0.760 | 0.530 |
| Anhui | 0.290 | 0.510 | 0.310 | 0.280 | 0.240 | 0.400 | 0.281 | 0.570 | 0.000 | 0.280 | 0.350 | 0.120 | 0.240 | 0.150 | 0.640 | 0.480 |
| Fujian | 0.350 | 0.460 | 0.360 | 0.330 | 0.240 | 0.600 | 0.275 | 0.690 | 0.440 | 0.370 | 0.270 | 0.330 | 0.420 | 0.540 | 0.790 | 0.120 |
| Jiangxi | 0.210 | 0.380 | 0.220 | 0.180 | 0.190 | 0.420 | 0.169 | 0.580 | 0.080 | 0.170 | 0.200 | 0.110 | 0.260 | 0.150 | 0.650 | 0.310 |
| Shandong | 0.810 | 1.000 | 0.840 | 0.880 | 0.530 | 0.520 | 0.700 | 0.700 | 0.170 | 0.360 | 0.400 | 0.270 | 0.360 | 0.440 | 0.080 | 0.290 |
| Henan | 0.490 | 0.800 | 0.520 | 0.510 | 0.290 | 0.340 | 0.379 | 0.580 | 0.040 | 0.190 | 0.200 | 0.060 | 0.240 | 0.180 | 0.640 | 0.520 |
| Hubei | 0.390 | 0.570 | 0.380 | 0.450 | 0.280 | 0.500 | 0.324 | 0.550 | 0.440 | 0.390 | 0.320 | 0.130 | 0.290 | 0.320 | 0.710 | 0.580 |
| Hunan | 0.370 | 0.560 | 0.350 | 0.380 | 0.230 | 0.420 | 0.342 | 0.600 | 0.170 | 0.380 | 0.270 | 0.180 | 0.250 | 0.210 | 0.710 | 0.070 |
| Guangdong | 1.000 | 0.670 | 1.000 | 1.000 | 1.000 | 0.690 | 1.000 | 0.830 | 0.780 | 0.660 | 0.440 | 0.380 | 0.390 | 0.520 | 0.620 | 0.630 |
| Guangxi | 0.190 | 0.350 | 0.210 | 0.190 | 0.130 | 0.320 | 0.166 | 0.280 | 0.010 | 0.090 | 0.100 | 0.090 | 0.160 | 0.100 | 0.730 | 0.720 |
| Hainan | 0.040 | 0.040 | 0.010 | 0.030 | 0.040 | 0.500 | 0.038 | 0.000 | 0.440 | 0.080 | 0.050 | 0.100 | 0.240 | 0.200 | 0.980 | 0.680 |
| Chongqing | 0.200 | 0.290 | 0.190 | 0.200 | 0.190 | 0.580 | 0.182 | 0.710 | 0.160 | 0.430 | 0.310 | 0.140 | 0.230 | 0.350 | 0.770 | 0.310 |
| Sichuan | 0.400 | 0.560 | 0.340 | 0.450 | 0.300 | 0.350 | 0.377 | 0.470 | 0.260 | 0.180 | 0.280 | 0.090 | 0.210 | 0.160 | 0.570 | 0.070 |
| Guizhou | 0.140 | 0.250 | 0.110 | 0.100 | 0.130 | 0.270 | 0.121 | 0.310 | 0.100 | 0.170 | 0.090 | 0.050 | 0.040 | 0.090 | 0.370 | 0.350 |
| Yunnan | 0.170 | 0.320 | 0.120 | 0.160 | 0.150 | 0.280 | 0.147 | 0.340 | 0.510 | 0.170 | 0.140 | 0.100 | 0.090 | 0.060 | 0.570 | 0.360 |
| Tibet | 0.000 | 0.000 | 0.000 | 0.000 | 0.000 | 0.000 | 0.000 | 0.580 | 0.260 | 0.000 | 0.000 | 0.090 | 0.110 | 0.110 | 1.000 | 0.550 |
| Shaanxi | 0.230 | 0.410 | 0.230 | 0.200 | 0.160 | 0.460 | 0.183 | 0.640 | 0.570 | 0.460 | 0.350 | 0.100 | 0.110 | 0.290 | 0.640 | 0.330 |
| Gansu | 0.070 | 0.070 | 0.050 | 0.080 | 0.060 | 0.270 | 0.069 | 0.470 | 0.460 | 0.130 | 0.180 | 0.010 | 0.000 | 0.000 | 0.690 | 0.540 |
| Qinghai | 0.010 | 0.040 | 0.020 | 0.010 | 0.010 | 0.390 | 0.012 | 0.590 | 0.450 | 0.070 | 0.090 | 0.050 | 0.070 | 0.150 | 0.850 | 0.420 |
| Ningxia | 0.020 | 0.030 | 0.030 | 0.010 | 0.020 | 0.480 | 0.019 | 0.670 | 0.560 | 0.330 | 0.170 | 0.060 | 0.130 | 0.220 | 0.720 | 0.120 |
| Xinjiang | 0.110 | 0.190 | 0.080 | 0.070 | 0.120 | 0.330 | 0.089 | 0.340 | 0.220 | 0.110 | 0.060 | 0.090 | 0.150 | 0.160 | 0.350 | 0.590 |

S2-7 RED Standardization Data in 2018

| **Scope of research** | V1 | V2 | V3 | V4 | V5 | V6 | V7 | V8 | V9 | V10 | V11 | V12 | V13 | V14 | V15 | V16 |
| --- | --- | --- | --- | --- | --- | --- | --- | --- | --- | --- | --- | --- | --- | --- | --- | --- |
| Beijing | 0.300 | 0.100 | 0.120 | 0.290 | 0.470 | 0.970 | 0.496 | 1.000 | 0.960 | 1.000 | 1.000 | 1.000 | 0.820 | 1.000 | 0.980 | 1.000 |
| Tianjin | 0.180 | 0.150 | 0.190 | 0.130 | 0.160 | 0.910 | 0.141 | 0.970 | 1.000 | 0.540 | 0.400 | 0.350 | 0.660 | 0.820 | 0.920 | 0.200 |
| Hebei | 0.360 | 0.590 | 0.390 | 0.410 | 0.280 | 0.440 | 0.288 | 0.560 | 0.220 | 0.180 | 0.190 | 0.100 | 0.240 | 0.150 | 0.000 | 0.280 |
| Shanxi | 0.160 | 0.080 | 0.120 | 0.170 | 0.170 | 0.480 | 0.153 | 0.800 | 0.660 | 0.250 | 0.130 | 0.050 | 0.140 | 0.130 | 0.290 | 0.290 |
| Inner Mongolia | 0.170 | 0.160 | 0.210 | 0.170 | 0.140 | 0.560 | 0.136 | 0.520 | 0.870 | 0.280 | 0.080 | 0.230 | 0.230 | 0.340 | 0.230 | 0.160 |
| Liaoning | 0.250 | 0.080 | 0.210 | 0.350 | 0.200 | 0.650 | 0.232 | 0.620 | 0.720 | 0.300 | 0.260 | 0.210 | 0.270 | 0.240 | 0.330 | 0.080 |
| Jilin | 0.140 | 0.200 | 0.180 | 0.180 | 0.090 | 0.460 | 0.099 | 0.640 | 0.750 | 0.200 | 0.090 | 0.030 | 0.230 | 0.220 | 0.750 | 0.210 |
| Heilongjiang | 0.160 | 0.150 | 0.100 | 0.220 | 0.090 | 0.510 | 0.104 | 0.120 | 0.890 | 0.170 | 0.100 | 0.000 | 0.230 | 0.110 | 0.510 | 0.000 |
| Shanghai | 0.330 | 0.100 | 0.220 | 0.310 | 0.580 | 1.000 | 0.460 | 1.000 | 0.740 | 0.740 | 0.660 | 1.000 | 1.000 | 0.950 | 0.960 | 0.170 |
| Jiangsu | 0.950 | 0.960 | 0.920 | 0.840 | 0.710 | 0.680 | 0.856 | 0.800 | 0.490 | 0.790 | 0.410 | 0.460 | 0.560 | 0.770 | 0.430 | 0.410 |
| Zhejiang | 0.570 | 0.570 | 0.580 | 0.630 | 0.540 | 0.660 | 0.556 | 0.840 | 0.600 | 0.700 | 0.390 | 0.680 | 0.860 | 0.620 | 0.760 | 0.560 |
| Anhui | 0.300 | 0.580 | 0.320 | 0.300 | 0.240 | 0.410 | 0.307 | 0.580 | 0.170 | 0.280 | 0.320 | 0.130 | 0.240 | 0.150 | 0.640 | 0.470 |
| Fujian | 0.360 | 0.500 | 0.370 | 0.350 | 0.230 | 0.610 | 0.310 | 0.690 | 0.640 | 0.380 | 0.260 | 0.330 | 0.420 | 0.550 | 0.790 | 0.110 |
| Jiangxi | 0.210 | 0.400 | 0.230 | 0.180 | 0.180 | 0.460 | 0.186 | 0.600 | 0.320 | 0.220 | 0.200 | 0.120 | 0.260 | 0.150 | 0.650 | 0.220 |
| Shandong | 0.780 | 1.000 | 0.830 | 0.850 | 0.530 | 0.530 | 0.620 | 0.700 | 0.040 | 0.350 | 0.320 | 0.270 | 0.350 | 0.410 | 0.080 | 0.260 |
| Henan | 0.490 | 0.820 | 0.530 | 0.510 | 0.300 | 0.360 | 0.389 | 0.580 | 0.110 | 0.170 | 0.190 | 0.070 | 0.230 | 0.170 | 0.640 | 0.470 |
| Hubei | 0.400 | 0.600 | 0.390 | 0.460 | 0.260 | 0.510 | 0.334 | 0.570 | 0.510 | 0.370 | 0.310 | 0.140 | 0.290 | 0.320 | 0.710 | 0.580 |
| Hunan | 0.360 | 0.590 | 0.350 | 0.390 | 0.220 | 0.440 | 0.345 | 0.600 | 0.150 | 0.420 | 0.260 | 0.190 | 0.250 | 0.200 | 0.710 | 0.370 |
| Guangdong | 1.000 | 0.680 | 1.000 | 1.000 | 1.000 | 0.700 | 1.000 | 0.820 | 0.710 | 0.710 | 0.430 | 0.390 | 0.390 | 0.510 | 0.620 | 0.630 |
| Guangxi | 0.200 | 0.360 | 0.200 | 0.200 | 0.120 | 0.340 | 0.170 | 0.290 | 0.160 | 0.070 | 0.080 | 0.080 | 0.170 | 0.090 | 0.730 | 0.660 |
| Hainan | 0.040 | 0.030 | 0.010 | 0.030 | 0.040 | 0.490 | 0.040 | 0.000 | 0.540 | 0.050 | 0.050 | 0.110 | 0.240 | 0.190 | 0.980 | 0.680 |
| Chongqing | 0.200 | 0.300 | 0.180 | 0.190 | 0.170 | 0.600 | 0.184 | 0.680 | 0.200 | 0.520 | 0.300 | 0.150 | 0.230 | 0.320 | 0.770 | 0.280 |
| Sichuan | 0.410 | 0.590 | 0.350 | 0.450 | 0.310 | 0.370 | 0.402 | 0.480 | 0.210 | 0.220 | 0.260 | 0.100 | 0.210 | 0.160 | 0.570 | 0.210 |
| Guizhou | 0.140 | 0.280 | 0.120 | 0.090 | 0.130 | 0.290 | 0.129 | 0.300 | 0.000 | 0.180 | 0.100 | 0.060 | 0.040 | 0.090 | 0.370 | 0.330 |
| Yunnan | 0.170 | 0.350 | 0.130 | 0.160 | 0.150 | 0.290 | 0.143 | 0.330 | 0.520 | 0.210 | 0.130 | 0.110 | 0.090 | 0.050 | 0.570 | 0.240 |
| Tibet | 0.000 | 0.000 | 0.000 | 0.000 | 0.000 | 0.000 | 0.000 | 0.580 | 0.390 | 0.000 | 0.000 | 0.120 | 0.120 | 0.110 | 1.000 | 0.470 |
| Shaanxi | 0.240 | 0.430 | 0.240 | 0.210 | 0.170 | 0.480 | 0.189 | 0.650 | 0.690 | 0.420 | 0.330 | 0.110 | 0.110 | 0.300 | 0.640 | 0.320 |
| Gansu | 0.070 | 0.060 | 0.050 | 0.070 | 0.050 | 0.290 | 0.068 | 0.470 | 0.420 | 0.100 | 0.160 | 0.020 | 0.000 | 0.000 | 0.690 | 0.490 |
| Qinghai | 0.010 | 0.040 | 0.030 | 0.010 | 0.000 | 0.410 | 0.012 | 0.560 | 0.560 | 0.070 | 0.060 | 0.060 | 0.070 | 0.150 | 0.850 | 0.400 |
| Ningxia | 0.020 | 0.030 | 0.030 | 0.010 | 0.020 | 0.490 | 0.019 | 0.650 | 0.410 | 0.330 | 0.170 | 0.070 | 0.130 | 0.210 | 0.720 | 0.040 |
| Xinjiang | 0.110 | 0.130 | 0.080 | 0.070 | 0.110 | 0.350 | 0.090 | 0.340 | 0.340 | 0.090 | 0.050 | 0.090 | 0.150 | 0.170 | 0.350 | 0.080 |

S2-8 RED Standardization Data in 2019

| **Scope of research** | V1 | V2 | V3 | V4 | V5 | V6 | V7 | V8 | V9 | V10 | V11 | V12 | V13 | V14 | V15 | V16 |
| --- | --- | --- | --- | --- | --- | --- | --- | --- | --- | --- | --- | --- | --- | --- | --- | --- |
| Beijing | 0.318 | 0.107 | 0.105 | 0.339 | 0.450 | 0.966 | 0.482 | 1.000 | 1.000 | 1.000 | 1.000 | 1.000 | 0.819 | 1.000 | 0.990 | 1.000 |
| Tianjin | 0.116 | 0.174 | 0.109 | 0.082 | 0.176 | 0.910 | 0.135 | 0.957 | 0.966 | 0.492 | 0.503 | 0.354 | 0.644 | 0.526 | 0.970 | 0.241 |
| Hebei | 0.313 | 0.620 | 0.287 | 0.290 | 0.283 | 0.443 | 0.288 | 0.578 | 0.178 | 0.095 | 0.226 | 0.112 | 0.244 | 0.097 | 0.046 | 0.379 |
| Shanxi | 0.144 | 0.086 | 0.165 | 0.148 | 0.171 | 0.489 | 0.132 | 0.802 | 0.663 | 0.078 | 0.144 | 0.054 | 0.139 | 0.108 | 0.215 | 0.517 |
| Inner Mongolia | 0.146 | 0.156 | 0.137 | 0.101 | 0.148 | 0.584 | 0.128 | 0.547 | 0.903 | 0.050 | 0.100 | 0.229 | 0.240 | 0.287 | 0.000 | 0.172 |
| Liaoning | 0.218 | 0.080 | 0.203 | 0.211 | 0.195 | 0.671 | 0.207 | 0.634 | 0.682 | 0.149 | 0.296 | 0.206 | 0.275 | 0.183 | 0.150 | 0.000 |
| Jilin | 0.094 | 0.159 | 0.082 | 0.082 | 0.072 | 0.496 | 0.091 | 0.539 | 0.765 | 0.102 | 0.167 | 0.032 | 0.225 | 0.101 | 0.620 | 0.379 |
| Heilongjiang | 0.111 | 0.159 | 0.082 | 0.115 | 0.084 | 0.550 | 0.098 | 0.000 | 0.864 | 0.090 | 0.137 | 0.000 | 0.227 | 0.051 | 0.376 | 0.241 |
| Shanghai | 0.341 | 0.103 | 0.242 | 0.357 | 0.558 | 1.000 | 0.451 | 1.000 | 0.668 | 0.529 | 0.622 | 0.995 | 1.000 | 0.933 | 0.946 | 0.207 |
| Jiangsu | 0.912 | 1.000 | 0.951 | 0.875 | 0.690 | 0.694 | 0.841 | 0.828 | 0.456 | 0.492 | 0.424 | 0.469 | 0.554 | 0.645 | 0.242 | 0.414 |
| Zhejiang | 0.572 | 0.609 | 0.574 | 0.630 | 0.549 | 0.678 | 0.552 | 0.871 | 0.605 | 0.525 | 0.400 | 0.681 | 0.859 | 0.504 | 0.678 | 0.586 |
| Anhui | 0.331 | 0.591 | 0.283 | 0.405 | 0.238 | 0.411 | 0.302 | 0.672 | 0.118 | 0.188 | 0.296 | 0.154 | 0.246 | 0.203 | 0.419 | 0.552 |
| Fujian | 0.382 | 0.509 | 0.411 | 0.430 | 0.228 | 0.610 | 0.308 | 0.750 | 0.622 | 0.278 | 0.252 | 0.342 | 0.422 | 0.535 | 0.706 | 0.241 |
| Jiangxi | 0.216 | 0.428 | 0.222 | 0.220 | 0.182 | 0.449 | 0.183 | 0.655 | 0.326 | 0.157 | 0.215 | 0.131 | 0.262 | 0.157 | 0.452 | 0.448 |
| Shandong | 0.648 | 0.877 | 0.580 | 0.675 | 0.507 | 0.500 | 0.612 | 0.698 | 0.032 | 0.176 | 0.308 | 0.265 | 0.346 | 0.277 | 0.060 | 0.310 |
| Henan | 0.489 | 0.868 | 0.456 | 0.538 | 0.307 | 0.356 | 0.423 | 0.642 | 0.113 | 0.130 | 0.202 | 0.076 | 0.235 | 0.155 | 0.450 | 0.345 |
| Hubei | 0.411 | 0.653 | 0.399 | 0.520 | 0.255 | 0.499 | 0.371 | 0.651 | 0.475 | 0.191 | 0.307 | 0.155 | 0.287 | 0.331 | 0.602 | 0.621 |
| Hunan | 0.359 | 0.632 | 0.304 | 0.377 | 0.224 | 0.419 | 0.336 | 0.621 | 0.068 | 0.164 | 0.284 | 0.207 | 0.245 | 0.200 | 0.509 | 0.517 |
| Guangdong | 1.000 | 0.776 | 1.000 | 1.000 | 1.000 | 0.697 | 1.000 | 0.841 | 0.804 | 0.432 | 0.432 | 0.400 | 0.390 | 0.411 | 0.385 | 0.655 |
| Guangxi | 0.184 | 0.401 | 0.131 | 0.176 | 0.128 | 0.338 | 0.166 | 0.323 | 0.176 | 0.047 | 0.088 | 0.089 | 0.172 | 0.064 | 0.638 | 0.552 |
| Hainan | 0.034 | 0.019 | 0.012 | 0.028 | 0.048 | 0.454 | 0.038 | 0.142 | 0.460 | 0.032 | 0.051 | 0.118 | 0.233 | 0.151 | 1.000 | 0.655 |
| Chongqing | 0.206 | 0.309 | 0.165 | 0.257 | 0.154 | 0.617 | 0.196 | 0.728 | 0.164 | 0.200 | 0.287 | 0.163 | 0.234 | 0.312 | 0.803 | 0.552 |
| Sichuan | 0.420 | 0.640 | 0.339 | 0.488 | 0.310 | 0.381 | 0.395 | 0.565 | 0.161 | 0.117 | 0.268 | 0.121 | 0.214 | 0.165 | 0.444 | 0.310 |
| Guizhou | 0.142 | 0.277 | 0.111 | 0.159 | 0.124 | 0.310 | 0.128 | 0.427 | 0.000 | 0.052 | 0.100 | 0.081 | 0.048 | 0.071 | 0.465 | 0.379 |
| Yunnan | 0.203 | 0.357 | 0.135 | 0.223 | 0.149 | 0.259 | 0.190 | 0.448 | 0.536 | 0.054 | 0.114 | 0.123 | 0.096 | 0.115 | 0.515 | 0.310 |
| Tibet | 0.000 | 0.000 | 0.000 | 0.000 | 0.000 | 0.000 | 0.000 | 0.659 | 0.168 | 0.000 | 0.000 | 0.151 | 0.141 | 0.101 | 0.980 | 0.448 |
| Shaanxi | 0.227 | 0.431 | 0.239 | 0.224 | 0.166 | 0.489 | 0.187 | 0.681 | 0.644 | 0.188 | 0.333 | 0.120 | 0.114 | 0.242 | 0.594 | 0.345 |
| Gansu | 0.066 | 0.064 | 0.056 | 0.069 | 0.051 | 0.296 | 0.065 | 0.496 | 0.477 | 0.049 | 0.167 | 0.032 | 0.000 | 0.000 | 0.613 | 0.414 |
| Qinghai | 0.012 | 0.039 | 0.018 | 0.004 | 0.005 | 0.444 | 0.009 | 0.569 | 0.526 | 0.036 | 0.073 | 0.067 | 0.079 | 0.120 | 0.914 | 0.690 |
| Ningxia | 0.027 | 0.011 | 0.029 | 0.015 | 0.016 | 0.532 | 0.016 | 0.690 | 0.337 | 0.089 | 0.154 | 0.079 | 0.137 | 0.140 | 0.782 | 0.172 |
| Xinjiang | 0.112 | 0.121 | 0.095 | 0.067 | 0.109 | 0.384 | 0.103 | 0.448 | 0.156 | 0.013 | 0.036 | 0.087 | 0.148 | 0.148 | 0.409 | 0.724 |

S2-9 RED Standardization Data in 2020

| **Scope of research** | V1 | V2 | V3 | V4 | V5 | V6 | V7 | V8 | V9 | V10 | V11 | V12 | V13 | V14 | V15 | V16 |
| --- | --- | --- | --- | --- | --- | --- | --- | --- | --- | --- | --- | --- | --- | --- | --- | --- |
| Beijing | 0.312 | 0.108 | 0.104 | 0.329 | 0.414 | 0.967 | 0.476 | 1.000 | 1.000 | 1.000 | 1.000 | 0.982 | 0.734 | 1.000 | 0.981 | 0.800 |
| Tianjin | 0.111 | 0.178 | 0.106 | 0.072 | 0.134 | 0.914 | 0.132 | 0.952 | 0.762 | 0.470 | 0.518 | 0.365 | 0.570 | 0.508 | 0.961 | 0.400 |
| Hebei | 0.312 | 0.638 | 0.291 | 0.303 | 0.284 | 0.454 | 0.289 | 0.584 | 0.245 | 0.092 | 0.245 | 0.136 | 1.000 | 0.097 | 0.438 | 0.440 |
| Shanxi | 0.146 | 0.098 | 0.168 | 0.152 | 0.163 | 0.500 | 0.131 | 0.752 | 0.679 | 0.078 | 0.153 | 0.081 | 0.131 | 0.118 | 0.496 | 0.600 |
| Inner Mongolia | 0.141 | 0.151 | 0.138 | 0.102 | 0.144 | 0.593 | 0.122 | 0.540 | 0.852 | 0.051 | 0.113 | 0.226 | 0.231 | 0.279 | 0.365 | 0.320 |
| Liaoning | 0.212 | 0.080 | 0.199 | 0.208 | 0.192 | 0.680 | 0.203 | 0.648 | 0.737 | 0.150 | 0.315 | 0.204 | 0.264 | 0.178 | 0.541 | 0.000 |
| Jilin | 0.095 | 0.173 | 0.086 | 0.078 | 0.068 | 0.502 | 0.088 | 0.508 | 0.835 | 0.096 | 0.172 | 0.050 | 0.212 | 0.115 | 0.793 | 0.480 |
| Heilongjiang | 0.107 | 0.164 | 0.076 | 0.110 | 0.073 | 0.558 | 0.094 | 0.000 | 0.941 | 0.078 | 0.167 | 0.000 | 0.216 | 0.051 | 0.652 | 0.480 |
| Shanghai | 0.339 | 0.115 | 0.243 | 0.385 | 0.537 | 1.000 | 0.449 | 1.000 | 0.930 | 0.564 | 0.627 | 1.000 | 0.912 | 0.943 | 0.946 | 0.360 |
| Jiangsu | 0.924 | 1.000 | 0.959 | 0.921 | 0.696 | 0.704 | 0.860 | 0.836 | 0.496 | 0.458 | 0.432 | 0.485 | 0.514 | 0.666 | 0.684 | 0.560 |
| Zhejiang | 0.575 | 0.642 | 0.574 | 0.656 | 0.553 | 0.680 | 0.569 | 0.876 | 0.881 | 0.509 | 0.424 | 0.697 | 0.801 | 0.506 | 0.789 | 0.720 |
| Anhui | 0.331 | 0.620 | 0.294 | 0.446 | 0.236 | 0.422 | 0.306 | 0.676 | 0.235 | 0.185 | 0.335 | 0.184 | 0.233 | 0.540 | 0.709 | 0.720 |
| Fujian | 0.382 | 0.504 | 0.398 | 0.453 | 0.225 | 0.616 | 0.323 | 0.764 | 0.582 | 0.271 | 0.273 | 0.354 | 0.391 | 0.165 | 0.815 | 0.320 |
| Jiangxi | 0.219 | 0.464 | 0.225 | 0.244 | 0.180 | 0.461 | 0.186 | 0.664 | 0.274 | 0.166 | 0.231 | 0.164 | 0.246 | 0.280 | 0.786 | 0.560 |
| Shandong | 0.649 | 0.907 | 0.586 | 0.722 | 0.499 | 0.510 | 0.617 | 0.716 | 0.268 | 0.191 | 0.333 | 0.278 | 0.312 | 0.147 | 0.546 | 0.600 |
| Henan | 0.479 | 0.904 | 0.450 | 0.551 | 0.311 | 0.368 | 0.419 | 0.624 | 0.000 | 0.119 | 0.229 | 0.080 | 0.214 | 0.295 | 0.711 | 0.560 |
| Hubei | 0.376 | 0.521 | 0.360 | 0.437 | 0.180 | 0.507 | 0.357 | 0.628 | 0.540 | 0.181 | 0.338 | 0.123 | 0.221 | 0.208 | 0.672 | 0.480 |
| Hunan | 0.363 | 0.680 | 0.312 | 0.393 | 0.219 | 0.430 | 0.331 | 0.604 | 0.228 | 0.164 | 0.310 | 0.234 | 0.232 | 0.411 | 0.759 | 0.760 |
| Guangdong | 1.000 | 0.832 | 1.000 | 1.000 | 1.000 | 0.717 | 1.000 | 0.844 | 0.826 | 0.412 | 0.465 | 0.422 | 0.364 | 0.065 | 0.628 | 0.840 |
| Guangxi | 0.185 | 0.417 | 0.128 | 0.180 | 0.118 | 0.345 | 0.170 | 0.352 | 0.081 | 0.035 | 0.089 | 0.105 | 0.166 | 0.153 | 0.804 | 0.720 |
| Hainan | 0.034 | 0.022 | 0.010 | 0.031 | 0.047 | 0.458 | 0.039 | 0.196 | 0.582 | 0.030 | 0.069 | 0.132 | 0.220 | 0.331 | 1.000 | 0.720 |
| Chongqing | 0.212 | 0.321 | 0.175 | 0.280 | 0.148 | 0.630 | 0.199 | 0.724 | 0.354 | 0.196 | 0.300 | 0.196 | 0.223 | 0.402 | 0.882 | 0.040 |
| Sichuan | 0.427 | 0.656 | 0.339 | 0.509 | 0.318 | 0.392 | 0.398 | 0.552 | 0.350 | 0.113 | 0.312 | 0.157 | 0.207 | 0.173 | 0.669 | 0.400 |
| Guizhou | 0.146 | 0.285 | 0.115 | 0.180 | 0.123 | 0.325 | 0.132 | 0.444 | 0.112 | 0.049 | 0.108 | 0.110 | 0.048 | 0.082 | 0.000 | 0.320 |
| Yunnan | 0.207 | 0.385 | 0.136 | 0.229 | 0.149 | 0.267 | 0.190 | 0.424 | 0.590 | 0.047 | 0.124 | 0.141 | 0.093 | 0.126 | 0.657 | 0.280 |
| Tibet | 0.000 | 0.000 | 0.000 | 0.000 | 0.000 | 0.000 | 0.000 | 0.696 | 0.600 | 0.000 | 0.000 | 0.222 | 0.158 | 0.128 | 0.994 | 0.680 |
| Shaanxi | 0.221 | 0.447 | 0.219 | 0.225 | 0.160 | 0.503 | 0.188 | 0.664 | 0.561 | 0.172 | 0.353 | 0.149 | 0.110 | 0.234 | 0.735 | 0.400 |
| Gansu | 0.065 | 0.070 | 0.055 | 0.073 | 0.051 | 0.308 | 0.065 | 0.480 | 0.445 | 0.043 | 0.159 | 0.060 | 0.000 | 0.000 | 0.831 | 0.520 |
| Qinghai | 0.010 | 0.027 | 0.016 | 0.003 | 0.006 | 0.455 | 0.009 | 0.564 | 0.659 | 0.027 | 0.077 | 0.097 | 0.074 | 0.117 | 0.941 | 1.000 |
| Ningxia | 0.025 | 0.010 | 0.029 | 0.014 | 0.016 | 0.546 | 0.017 | 0.668 | 0.617 | 0.097 | 0.171 | 0.102 | 0.132 | 0.149 | 0.893 | 0.280 |
| Xinjiang | 0.109 | 0.145 | 0.089 | 0.059 | 0.099 | 0.388 | 0.099 | 0.436 | 0.599 | 0.008 | 0.035 | 0.082 | 0.138 | 0.138 | 0.583 | 0.880 |

S2-10 RED Standardization Data in 2021

| **Scope of research** | V1 | V2 | V3 | V4 | V5 | V6 | V7 | V8 | V9 | V10 | V11 | V12 | V13 | V14 | V15 | V16 |
| --- | --- | --- | --- | --- | --- | --- | --- | --- | --- | --- | --- | --- | --- | --- | --- | --- |
| Beijing | 0.318 | 0.113 | 0.122 | 0.324 | 0.412 | 0.966 | 0.467 | 0.996 | 1.000 | 1.000 | 1.000 | 0.981 | 0.807 | 1.000 | 0.976 | 0.481 |
| Tianjin | 0.111 | 0.182 | 0.112 | 0.068 | 0.139 | 0.916 | 0.124 | 0.948 | 0.739 | 0.537 | 0.551 | 0.366 | 0.610 | 0.496 | 0.951 | 0.296 |
| Hebei | 0.312 | 0.623 | 0.309 | 0.293 | 0.285 | 0.466 | 0.277 | 0.576 | 0.232 | 0.093 | 0.255 | 0.126 | 0.249 | 0.090 | 0.176 | 0.519 |
| Shanxi | 0.170 | 0.108 | 0.222 | 0.160 | 0.189 | 0.509 | 0.131 | 0.762 | 0.702 | 0.089 | 0.133 | 0.078 | 0.143 | 0.168 | 0.483 | 0.815 |
| Inner Mongolia | 0.156 | 0.164 | 0.172 | 0.098 | 0.154 | 0.600 | 0.114 | 0.541 | 0.890 | 0.048 | 0.100 | 0.220 | 0.255 | 0.322 | 0.000 | 0.259 |
| Liaoning | 0.208 | 0.084 | 0.204 | 0.207 | 0.184 | 0.687 | 0.193 | 0.623 | 0.703 | 0.159 | 0.309 | 0.193 | 0.287 | 0.164 | 0.237 | 0.074 |
| Jilin | 0.090 | 0.190 | 0.081 | 0.079 | 0.067 | 0.508 | 0.085 | 0.502 | 0.859 | 0.117 | 0.181 | 0.041 | 0.229 | 0.097 | 0.756 | 0.444 |
| Heilongjiang | 0.104 | 0.172 | 0.077 | 0.109 | 0.078 | 0.552 | 0.092 | 0.000 | 0.981 | 0.092 | 0.167 | 0.000 | 0.238 | 0.042 | 0.561 | 0.481 |
| Shanghai | 0.339 | 0.125 | 0.235 | 0.398 | 0.544 | 1.000 | 0.449 | 1.000 | 0.917 | 0.579 | 0.634 | 1.000 | 1.000 | 0.917 | 0.937 | 0.667 |
| Jiangsu | 0.940 | 1.000 | 0.989 | 0.966 | 0.706 | 0.708 | 0.863 | 0.831 | 0.489 | 0.519 | 0.432 | 0.494 | 0.567 | 0.664 | 0.613 | 0.741 |
| Zhejiang | 0.511 | 0.676 | 0.597 | 0.655 | 0.579 | 0.684 | 0.573 | 0.879 | 0.904 | 0.491 | 0.498 | 0.714 | 0.879 | 0.497 | 0.717 | 0.704 |
| Anhui | 0.330 | 0.646 | 0.287 | 0.476 | 0.236 | 0.432 | 0.306 | 0.671 | 0.238 | 0.229 | 0.339 | 0.192 | 0.256 | 0.196 | 0.619 | 0.741 |
| Fujian | 0.387 | 0.509 | 0.391 | 0.451 | 0.228 | 0.628 | 0.322 | 0.753 | 0.619 | 0.349 | 0.272 | 0.359 | 0.435 | 0.146 | 0.776 | 0.444 |
| Jiangxi | 0.226 | 0.492 | 0.235 | 0.263 | 0.187 | 0.472 | 0.190 | 0.667 | 0.324 | 0.167 | 0.228 | 0.165 | 0.268 | 0.531 | 0.700 | 0.630 |
| Shandong | 0.659 | 0.910 | 0.602 | 0.759 | 0.509 | 0.519 | 0.628 | 0.693 | 0.211 | 0.259 | 0.336 | 0.275 | 0.346 | 0.171 | 0.370 | 0.593 |
| Henan | 0.457 | 0.893 | 0.414 | 0.543 | 0.298 | 0.377 | 0.409 | 0.597 | 0.000 | 0.133 | 0.240 | 0.071 | 0.225 | 0.277 | 0.631 | 0.407 |
| Hubei | 0.391 | 0.601 | 0.345 | 0.478 | 0.221 | 0.522 | 0.371 | 0.606 | 0.530 | 0.219 | 0.331 | 0.136 | 0.252 | 0.120 | 0.706 | 0.556 |
| Hunan | 0.356 | 0.697 | 0.311 | 0.410 | 0.219 | 0.438 | 0.330 | 0.602 | 0.211 | 0.198 | 0.321 | 0.230 | 0.253 | 0.311 | 0.716 | 0.815 |
| Guangdong | 1.000 | 0.837 | 1.000 | 1.000 | 1.000 | 0.722 | 1.000 | 0.835 | 0.864 | 0.412 | 0.477 | 0.435 | 0.401 | 0.191 | 0.483 | 0.741 |
| Guangxi | 0.189 | 0.429 | 0.131 | 0.178 | 0.114 | 0.351 | 0.167 | 0.307 | 0.079 | 0.049 | 0.082 | 0.100 | 0.182 | 0.393 | 0.761 | 0.741 |
| Hainan | 0.036 | 0.032 | 0.011 | 0.039 | 0.051 | 0.462 | 0.042 | 0.169 | 0.622 | 0.059 | 0.071 | 0.135 | 0.245 | 0.063 | 1.000 | 0.519 |
| Chongqing | 0.212 | 0.327 | 0.171 | 0.303 | 0.149 | 0.640 | 0.200 | 0.710 | 0.353 | 0.232 | 0.304 | 0.202 | 0.246 | 0.157 | 0.863 | 0.593 |
| Sichuan | 0.424 | 0.660 | 0.339 | 0.538 | 0.328 | 0.403 | 0.399 | 0.554 | 0.333 | 0.118 | 0.320 | 0.160 | 0.227 | 0.161 | 0.601 | 0.333 |
| Guizhou | 0.142 | 0.263 | 0.115 | 0.187 | 0.126 | 0.336 | 0.128 | 0.407 | 0.104 | 0.052 | 0.104 | 0.114 | 0.053 | 0.065 | 0.725 | 0.000 |
| Yunnan | 0.205 | 0.382 | 0.142 | 0.229 | 0.149 | 0.274 | 0.184 | 0.390 | 0.611 | 0.046 | 0.123 | 0.149 | 0.102 | 0.114 | 0.560 | 0.259 |
| Tibet | 0.000 | 0.000 | 0.000 | 0.000 | 0.000 | 0.000 | 0.000 | 0.667 | 0.608 | 0.000 | 0.000 | 0.264 | 0.203 | 0.108 | 0.998 | 0.704 |
| Shaanxi | 0.229 | 0.411 | 0.246 | 0.218 | 0.184 | 0.513 | 0.183 | 0.658 | 0.548 | 0.182 | 0.333 | 0.145 | 0.122 | 0.240 | 0.700 | 0.370 |
| Gansu | 0.066 | 0.083 | 0.059 | 0.074 | 0.057 | 0.317 | 0.063 | 0.433 | 0.440 | 0.060 | 0.160 | 0.052 | 0.000 | 0.000 | 0.778 | 0.407 |
| Qinghai | 0.011 | 0.029 | 0.017 | 0.003 | 0.008 | 0.463 | 0.007 | 0.554 | 0.608 | 0.038 | 0.082 | 0.084 | 0.080 | 0.110 | 0.929 | 1.000 |
| Ningxia | 0.020 | 0.016 | 0.033 | 0.012 | 0.018 | 0.559 | 0.014 | 0.658 | 0.653 | 0.134 | 0.204 | 0.095 | 0.144 | 0.153 | 0.875 | 0.148 |
| Xinjiang | 0.116 | 0.167 | 0.100 | 0.064 | 0.101 | 0.392 | 0.096 | 0.372 | 0.686 | 0.021 | 0.031 | 0.082 | 0.153 | 0.150 | 0.440 | 0.926 |
